# ER stress sensor PERK promotes T cell pathogenicity in GVHD by regulating ER-associated degradation

**DOI:** 10.1172/JCI190958

**Published:** 2025-09-30

**Authors:** Qiao Cheng, Hee-Jin Choi, Yongxia Wu, Xiaohong Yuan, Allison Pugel, Linlu Tian, Michael Hendrix, Denggang Fu, Reza Alimohammadi, Chen Liu, Xue-Zhong Yu

**Affiliations:** 1 Department of Microbiology & Immunology and; 2 Cancer Center, Medical College of Wisconsin, Milwaukee, Wisconsin, USA.; 3 Department of Pathology, Yale University, New Haven, Connecticut, USA.; 4 Department of Medicine, Medical College of Wisconsin, Milwaukee, Wisconsin, USA.

**Keywords:** Immunology, Inflammation, Bone marrow transplantation

## Abstract

Endoplasmic reticulum (ER) stress through IRE1/XBP1 is implicated in the onset and progression of graft-versus-host disease (GVHD), but the role of the ER stress sensor PERK in T cell allogeneic responses and GVHD remains unexplored. Here, we report that PERK is a key regulator in T cell allogeneic response and GVHD induction. PERK augments GVHD through increasing Th1 and Th17 population, while reducing Treg differentiation by activating the Nrf2 pathway. Genetic deletion or selective inhibition of PERK pharmacologically reduces GVHD while preserving graft-versus-leukemia (GVL) activity. At the cellular level, PERK positively regulates CD4^+^ T cell pathogenicity while negatively regulating CD8^+^ T cell pathogenicity in the induction of GVHD. At the molecular level, PERK interacts with SEL1L and regulates SEL1L expression, leading to augmented T cell allogeneic responses and GVHD development. In vivo, PERK deficiency in donor T cells alleviates GVHD through ER-associated degradation. Furthermore, pharmacological inhibition of PERK with AMG44 significantly suppresses the severity of GVHD induced by murine or human T cells. In summary, our findings validate PERK as a potential therapeutic target for the prevention of GVHD while preserving GVL responses, and uncover the mechanism by which PERK differentially regulates CD4^+^ versus CD8^+^ T cell allogeneic and antitumor responses.

## Abstract 2

PERK regulates T cell allogeneic responses through ER-associated degradation and can be a novel potential target for alleviating GVHD while preserving the GVL responses.

## Graphical abstract

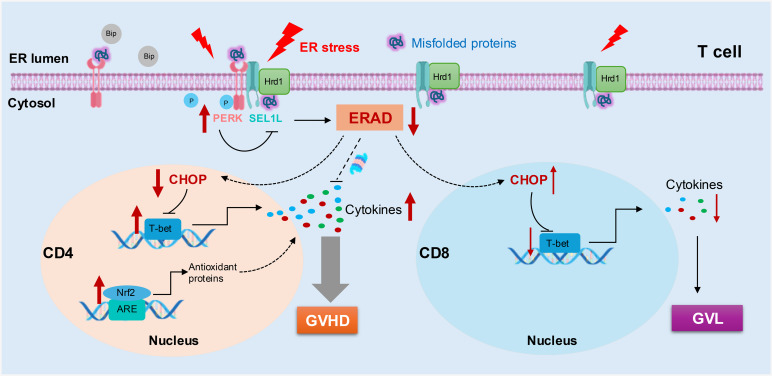

## Introduction

Acute graft-versus-host disease (GVHD) is a major complication that leads to the morbidity or mortality of patients with hematological malignancies receiving allogeneic hematopoietic cell transplantation (1, 2). GVHD is characterized by excessive activation of donor T cells and high levels of inflammatory cytokines. Endoplasmic reticulum (ER) stress through the IRE1/XBP1 pathway has been reported to increase upon GVHD onset, correlating with the severity of GVHD (3). While ER stress is an emerging regulator in GVHD development (4), the role of the ER stress sensor PERK in T cell allogeneic responses and GVHD pathogenicity is not clear.

An accumulation of unfolded or misfolded proteins in the ER leads to ER stress response. Activation of unfolded protein responses (UPRs) controls the quality of the proteome pool in ER-stressed cells and maintains intracellular protein homeostasis (5). UPR is involved in the development and effector function of diverse immune cells, including T cells (6–8), dendritic cells (9–11), macrophages (12), and myeloid cell–driven immunosuppressive cells (13). Thus, targeting UPR is becoming a promising strategy in managing human diseases (14, 15). UPR signaling includes 3 primary regulators: inositol-requiring enzyme-1α (IRE1α), activating transcription factor-6 (ATF6), and protein kinase R–like endoplasmic reticulum kinase (PERK), encoded by Eif2ak3 (16). Recently, IRE1α-mediated X-box–binding protein-1 (XBP1) signaling was reported to promote GVHD (9, 17), but the mechanism of how the ER stress sensors PERK and XBP1 regulate T cell allogeneic response and GVHD induction is essentially uncharacterized.

PERK resides in the ER lumen and includes 3 domains, the luminal domain, transmembrane domain, and cytosolic domain (18, 19). C/EBP-homologous protein (CHOP) induced by ER stress was reported to suppress antitumor CD8^+^ T cell immunity through inducing ATF4 and directly repressing T-bet expression, a master regulator of T cell effector function (20). Inhibition of PERK promotes antitumor immunity through suppressing immunosuppressive capacity of macrophages (12). Tumor-driven PERK was reported to promote immune evasion through regulating SEC61β-induced paraptosis and type I interferons (7). However, the function of PERK regulating CD4^+^ T cells is not thoroughly described. ER-associated protein degradation (ERAD) is a cellular pathway that targets misfolded proteins of ER for ubiquitination and subsequent degradation via proteasome (21). The SEL1L/HRD1 axis as a key component of the ERAD signaling pathway was reported to be involved in Th1/Th17 differentiation in mice with experimental autoimmune encephalomyelitis (EAE) (22). In addition, SEL1L has been reported to control the survival of CD8^+^ T cells (23) and is required for the function and memory formation of CD8^+^ T cells (24). However, the role of SEL1L in T cell allogeneic response and GVHD induction has not been elucidated.

In the current study, we observed that genetic ablation of PERK reduced T cell allogeneic responses and GVHD induction, while promoting T cell antitumor immunity. Thus, PERK appears to play a distinct role in regulating T cell allogeneic versus antitumor response. Our findings provide evidence that PERK can serve as a potential therapeutic target for the prevention of GVHD while preserving graft-versus-leukemia (GVL) responses. The study also elucidates the mechanism by which PERK distinctly regulates CD4^+^ versus CD8^+^ T cell allogeneic and antitumor responses.

## Results

### Allogeneic stimulation activates ER stress signaling in T cells.

**Figure 1:**
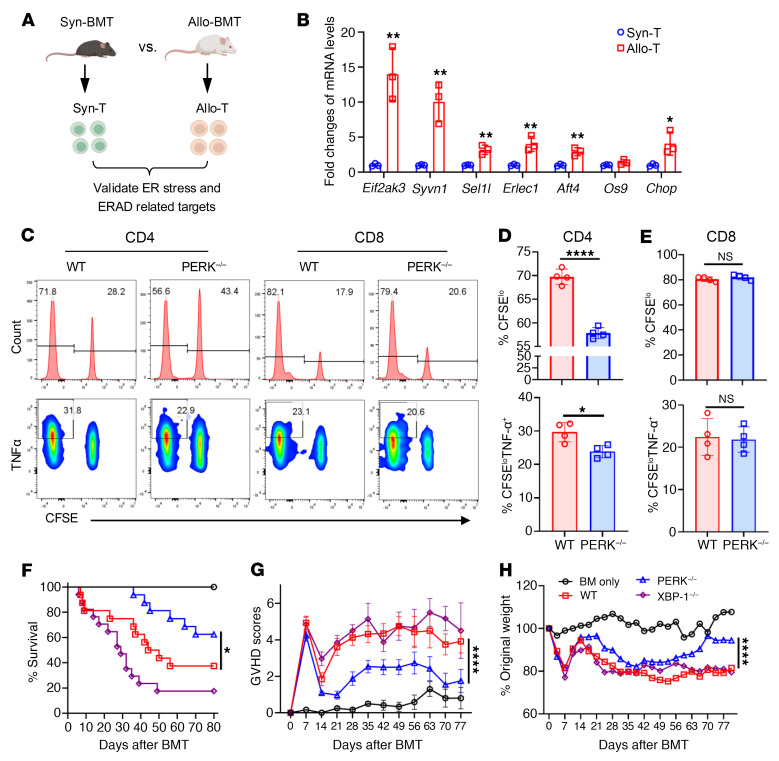
PERK but not XBP1 positively regulates T cell allogeneic responses and GVHD. (**A**) T cells (1.25 × 10^6^) isolated from normal B6 (Ly5.2) donors plus BM cells (4 × 10^6^) from Rag1-KO donors were transferred into lethally irradiated WT B6 (Ly5.1, syngeneic) and BALB/c (allogeneic) recipients; *n* = 5 per group. (**B**) T cells were isolated from spleens of B6 (Ly5.1) and BALB/c recipients on day 14 after BMT, and total RNA was extracted. mRNA levels of *Eif2ak3*, *Syvn1*, *Sel1l*, *Erlec1*, *Atf4*, *Os9*, *Chop*, and *Actin* were analyzed by quantitative real-time PCR. (**C**) T cells isolated from WT and PERK-cKO mice were labeled with CFSE and stimulated with allogeneic APCs (TCD-splenocytes) from BDF1 mice for 4 days. The proliferation (CFSE^lo^) of CD4^+^ or CD8^+^ T cells and the levels of proinflammatory cytokines in CD4^+^ or CD8^+^ T cells were analyzed by flow cytometry. (**D**) Percentages of CFSE^lo^CD4^+^ and CFSE^lo^TNF-α^+^CD4^+^ T cells among gated H2K^d–^CD4^+^ T cells are shown. (**E**) Percentages of CFSE^lo^CD8^+^ and CFSE^lo^TNF-α^+^CD8^+^ T cells among gated H2K^d–^CD8^+^ T cells are shown. (**F**–**H**) TCD-BM cells (5 × 10^6^) alone or together with T cells (1.25 × 10^6^) from WT B6 or PERK-cKO or XBP1-cKO donors were transferred into lethally irradiated BALB/c recipients. Survival (**F**), GVHD scores (**G**), and body weight (**H**) of BALB/c recipients were monitored through 80 days after BMT; *n* = 5 for BM only, *n* = 16 for WT or PERK-cKO or XBP1-cKO combined from 2 replicate experiments. Log-rank (Mantel-Cox) test (**F**) and non-parametric Mann-Whitney *U* test (**G** and **H**) were used to compare groups. Data in **B**, **D**, and **E** are represented as mean ± SD; significance was determined using a 2-tailed unpaired Student’s *t* test. *P < 0.05, **P < 0.01, ****P < 0.0001.

To investigate whether ER stress is involved in allogeneic T cell response, we established syngeneic and murine allogeneic bone marrow transplantation (BMT) models (Figure 1A). T cell allogeneic responses were increased in the recipients after allogeneic BMT (Supplemental Figure 1, A–C; supplemental material available online with this article; https://doi.org/10.1172/JCI190958DS1), and we found that *Eif2ak3* (encoding PERK), *Syvn1*, *Sel1l*, *Erlec1*, *Atf4*, and *Chop* were significantly increased in the T cells after allogeneic response (Figure 1B). In vitro, we verified that phosphorylated PERK (p-PERK) and XBP1s increased significantly in CD4^+^ and CD8^+^ T cells after allogeneic stimulation (Supplemental Figure 1, D and E). These data suggest that ER stress signaling may play an essential role in allogeneic T cell response.

### PERK promotes allogeneic responses of CD4^+^ T cells and exacerbates GVHD.

To further evaluate how PERK and XBP1 affect T cell response to alloantigen in vivo, we used conditional-knockout (cKO) mice on C57BL/6 (B6) background that are specifically deficient in PERK or XBP1 in their T cells, respectively (Supplemental Figure 2A). To assess whether PERK or XBP1 impacts T cell development, we analyzed the frequency of both CD4^+^ and CD8^+^ T cells, CD44^+^CD62L^–^ (effector memory), CD44^+^CD62L^+^ (central memory), and CD44^–^CD62L^+^ (naive), in PERK- or XBP1-cKO mice. There was no significant difference in the percentages of CD4^+^ and CD8^+^ T cells in PERK- or XBP1-deficient mice compared with wild-type (WT) mice (Supplemental Figure 2, B–D). We further demonstrated that PERK or XBP1 deficiency did not significantly affect the percentages of naive, effector memory, or central memory CD4^+^ and CD8^+^ T cells in comparison with WT mice (Supplemental Figure 2, E–G). Thus, both PERK and XBP1 do not affect the development of T cells or their phenotypes at homeostatic condition.

To determine whether PERK regulates T cell allogeneic responses, T cells isolated from WT and PERK-cKO mice were labeled with CFSE, and then cocultured with T cell–depleted (TCD) splenocytes as allogeneic antigen-presenting cells (APCs). PERK deficiency markedly reduced the proliferation and activation of CD4^+^ but not CD8^+^ T cells as reflected by lower percentage of CFSE^lo^ cells and reduced TNF-α levels (Figure 1, C–E). Therefore, PERK preferentially promotes allogeneic responses of CD4^+^ T cells in vitro.

To assess whether PERK or XBP1 regulates T cell–driven GVHD, we used an MHC-mismatched murine BMT model, B6 → BALB/c. As compared with WT T cells, PERK-deficient T cells induced significantly milder GVHD in allogeneic recipients reflected by better survival (Figure 1F), reduced clinical scores (Figure 1G), and body weight loss (Figure 1H). However, XBP1-deficient T cells induced severe GVHD similarly to WT T cells (Figure 1, F–H). Consistently, the recipients of PERK-deficient donor T cells had significantly improved thymocyte recovery and B cell reconstitution as compared with those of WT or XBP1-deficient T cells (Supplemental Figure 3, A and B). Taken together, these results suggest that PERK promotes T cell pathogenicity in the induction of GVHD in mice.

### PERK deficiency in donor T cells alleviates gut GVHD through reducing Th1 and Th17 subsets and increasing Treg generation.

**Figure 2:**
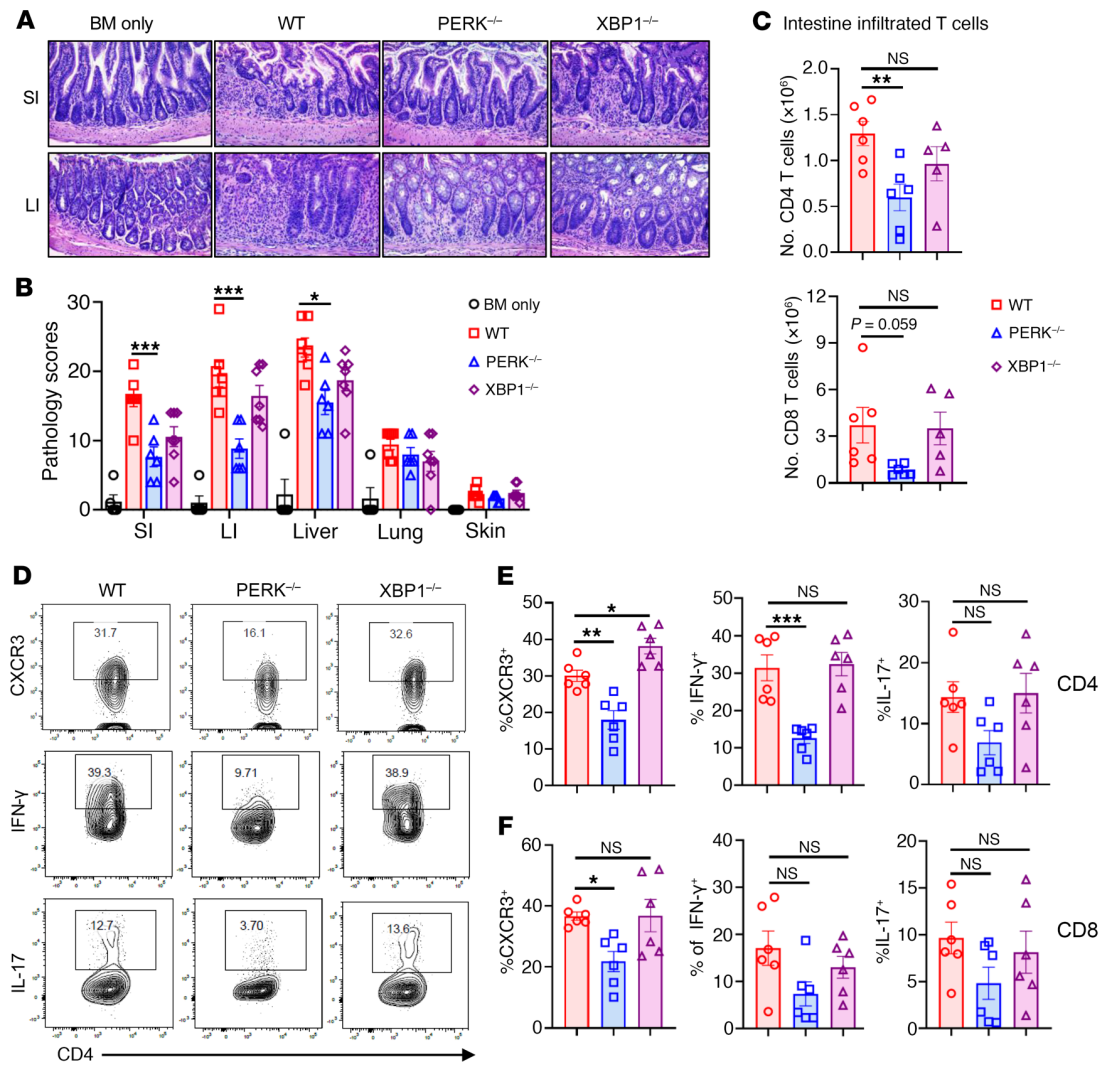
PERK-deficient T cells have reduced ability to induce GVHD and differentiate to Th1 and Th17 populations. Lethally irradiated BALB/c mice (900 cGy) were injected with TCD-BM cells (5 × 10^6^) alone or together with T cells (1.25 × 10^6^) isolated from WT B6, PERK-cKO, or XBP1-cKO donors; *n* = 5–7 per group. (**A**) Pathology of small intestine (SI) and large intestine (LI) of BALB/c recipients was analyzed on the tissues with H&E staining. Original magnification: ×200. (**B**) Pathology scores of small and large intestines, liver, lung, and skin are displayed. (**C**) Absolute number of infiltrated CD4^+^ or CD8^+^ T cells in intestine was analyzed on day 14 after BMT. (**D**) CXCR3^+^CD4^+^, IFN-γ^+^CD4^+^, and IL-17^+^CD4^+^ T cells in intestine were analyzed by flow cytometry. (**E**) Percentages of CXCR3^+^CD4^+^, IFN-γ^+^CD4^+^, and IL-17^+^CD4^+^ T cells among gated H2K^b+^CD4^+^ T cells are displayed. (**F**) Percentages of CXCR3^+^CD8^+^, IFN-γ^+^CD8^+^, and IL-17^+^CD8^+^ T cells among gated H2K^b+^CD8^+^ T cells are displayed. Data in **B**, **C**, **E**, and **F** are represented as mean ± SD; significance was determined using a 1-way ANOVA test. *P < 0.05, **P < 0.01, ***P < 0.001.

To explore how PERK regulates T cell–mediated GVHD, we used the same allogeneic BMT model (B6 → BALB/c) as described above. PERK-deficient T cells induced milder GVHD reflected by less body weight loss of the recipients when compared with WT T cells, which is consistent with long-term BMT experiments (Supplemental Figure 4, A and B). Two weeks after BMT, pathological analyses confirmed that the recipients of PERK-deficient T cells had significantly reduced injuries in the liver, as well as the small and large intestines, as compared with those of WT or XBP1-deficient T cells (Figure 2, A and B). We examined donor T cells in recipient intestines and found that infiltrated CD4^+^ and CD8^+^ T cells were decreased in recipients receiving PERK-deficient T cells as compared with those in the recipients of WT XBP1-deficient T cells (Figure 2C). We further observed that only PERK-deficient T cells significantly reduced CXCR3 expression levels on donor CD4^+^ and CD8^+^ T cells. PERK deficiency also significantly reduced IFN-γ and substantially decreased IL-17 production by donor CD4^+^ but not CD8^+^ T cells (Figure 2, D–F). We also observed that PERK deficiency significantly decreased CXCR3, TNF-α, and IL-17A in donor CD4^+^ but not CD8^+^ T cells in recipient small intestine (Supplemental Figure 4, C–E). Based on these findings, PERK may promote allogeneic T cell differentiation toward Th1 and Th17 cells that are major pathogenic subsets for GVHD induction.

In addition, we found that the frequency and absolute number of CD4^+^Foxp3^+^ T cells were increased among PERK-deficient T cells as compared with WT or XBP1-deficient counterparts (Supplemental Figure 5, A–C). Consistently, PERK deficiency promoted T cell differentiation toward CD25^+^Foxp3^+^ Tregs under Treg polarization condition in vitro (Supplemental Figure 5, D and E). Nrf2 is known to be a key regulator for Treg differentiation (25). We therefore analyzed the levels of Nrf2 in Tregs and found that PERK deficiency significantly reduced the levels of Nrf2 (Supplemental Figure 5F). To further assess whether PERK regulated Treg differentiation through Nrf2, we activated Nrf2 using sulforaphane (SFN) and observed that SFN treatment reversed the heightened Treg differentiation as well as Helios expression due to PERK deficiency (Supplemental Figure 5, G–I). Thus, PERK appears to regulate Treg differentiation through the Nrf2 signaling pathway.

### PERK deficiency or inhibition does not impact T cell–mediated GVL effect.

**Figure 3:**
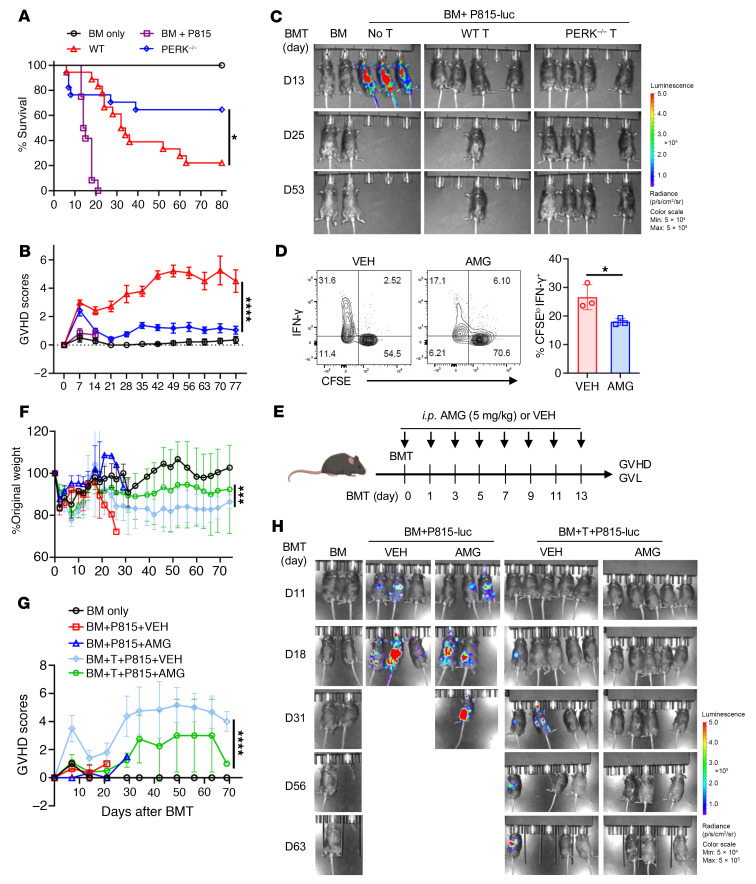
PERK deficiency in donor T cells does not impact their GVL activity while reducing ability to induce GVHD. (**A**–**C**) Lethally irradiated BDF1 mice were injected with TCD-BM cells (5 × 10^6^) alone or together with luciferase-expressing P815 (P815-luc) cells (5,000) with or without CD25-removed T cells (3 × 10^6^) from WT or PERK-cKO donors. Survival (**A**) and GVHD scores (**B**) were monitored through 80 days after BMT. Tumor growth in BDF1 recipients was monitored using bioluminescent imaging (BLI) (**C**); *n* = 7 for BM only, *n* = 12 for BM with P815, *n* = 16–17 for WT or PERK-cKO. (**D**) T cells isolated from WT B6 mice were labeled with CFSE, stimulated with allogeneic APCs from BALB/c mice for 4 days, and treated with or without AMG44 (10 μM); CFSE^lo^IFN-γ^+^CD4^+^ T cells were analyzed using flow cytometry. Percentage of CFSE^lo^IFN-γ^+^CD4^+^ T cells among gated H2K^b+^CD4^+^ T cells is displayed. (**E**–**H**) Lethally irradiated BDF1 mice (1,200 cGy) were injected with TCD-BM cells (5 × 10^6^) alone or together with P815-luc cells (5,000) and with or without CD25-removed T cells (3 × 10^6^) from normal B6 mice. Vehicle (VEH) or AMG44 (5 mg/kg) was injected intraperitoneally (i.p.) into BDF1 recipients every other day for 2 weeks after BMT (**E**). Body weight loss (**F**) and GVHD scores (**G**) were monitored through 80 days after BMT. Tumor growth in BDF1 recipients was monitored using BLI (**H**); *n* = 2 for BM only, *n* = 3 for BM and P815 with VEH or AMG, *n* = 5 for BM+T+P815 with VEH or AMG. Log-rank (Mantel-Cox) test (**A**) and non-parametric Mann-Whitney *U* test (**B**, **F**, and **G**) were used to compare groups. Data in **D** are represented as mean ± SD with biological replicates; significance was determined using a 2-tailed unpaired Student’s *t* test. *P < 0.05, ***P < 0.001, ****P < 0.0001.

The data presented so far indicate that PERK but not XBP1 impacts T cell allogeneic response and GVHD induction, and we thus focused the rest of the study on PERK. Because allogeneic hematopoietic cell transplantation is primarily applied as a therapeutic approach to treat hematological malignancies, such as leukemia, we wanted to evaluate the impact of PERK on T cell–mediated GVL activity. To this end, CD25-depleted T cells isolated from WT and PERK-cKO mice were injected into BDF1 mice along with luciferase-transduced host-type mastocytoma (P815) cells. Consistently, the recipients of PERK-deficient T cells had milder GVHD than those of WT T cells, reflected by improved survival and decreased clinical scores (Figure 3, A and B). Furthermore, pathological analyses confirmed that the recipients of PERK-deficient T cells had significantly reduced injuries in the liver, lung, and small intestines when compared with those of WT T cells (Supplemental Figure 6, A and B). More importantly, PERK deficiency in T cells preserved GVL activity in this haploidentical BMT model (Figure 3C). In addition, PERK-deficient donor T cells exhibited intact GVL activity against mixed-lineage leukemia (MLL) but induced milder GVHD reflected by prolonged survival in recipients after MHC-mismatched BMT (Supplemental Figure 7, A and D). These results indicate that PERK deficiency preserves GVL effect while reducing GVHD.

For translational purposes, we next evaluated the effect of ER stress inhibition in allogeneic response in vitro by testing an ER stress inhibitor, tauroursodeoxycholic acid (TUDCA). TUDCA is a naturally occurring hydrophilic bile acid, which has been shown to attenuate ER stress, prevent UPR dysfunction, and stabilize mitochondria (26). TUDCA is FDA-approved for treating biliary cirrhosis. Recent studies show that TUDCA has additional beneficial effects in neurodegenerative diseases (27), osteoarthritis (28), vascular diseases (29), and diabetes (30). We first tested the effect of TUDCA in T cell responses in vitro. Consistent with the genetic model, we observed that TUDCA significantly decreased CD4^+^ T cell proliferation (CFSE dilution) and activation (IFN-γ production) upon allogeneic stimulation in vitro (Supplemental Figure 8A). Furthermore, TUDCA treatment significantly prolonged recipient survival (Supplemental Figure 8B) and reduced clinical scores (Supplemental Figure 8C). Consistent with PERK deficiency in T cells, TUDCA treatment also preserved the GVL activity (Supplemental Figure 8D). Given that TUDCA inhibits both PERK and XBP1, we attempted to determine how much the contribution of TUDCA treatment to GVHD alleviation was due to inhibition of XBP1 versus PERK. To do so, we transplanted XBP1-deficient T cells and treated the recipients with TUDCA. As shown in Figure 1, F–H, XBP1-deficient T cells induced similarly severe GVHD as compared with WT cells. The treatment with TUDCA was effective in preventing GVHD while preserving the GVL activity induced by either type of T cells (Supplemental Figure 8, E–G). Taken together, these findings suggest that targeting of ER stress by TUDCA can preserve GVL activity while attenuating GVHD primarily through inhibiting PERK.

AMG44 is a highly selective PERK inhibitor with an IC_50_ of 6 nM that has been tested in several recent studies (20, 31, 32). We therefore tested AMG44 and found that the PERK-specific inhibitor significantly reduced proliferation and IFN-γ production of CD4^+^ T cells in response to alloantigen stimulation in vitro (Figure 3D). Furthermore, treatment with AMG44 significantly attenuated GVHD severity (Figure 3, E–G) while preserving the GVL effect (Figure 3H). Thus, our data indicate PERK serving as a potential therapeutic target for the prevention of GVHD while maintaining GVL activity after allogeneic BMT.

### PERK differentially regulates CD4^+^ and CD8^+^ T cell allogeneic responses.

**Figure 4:**
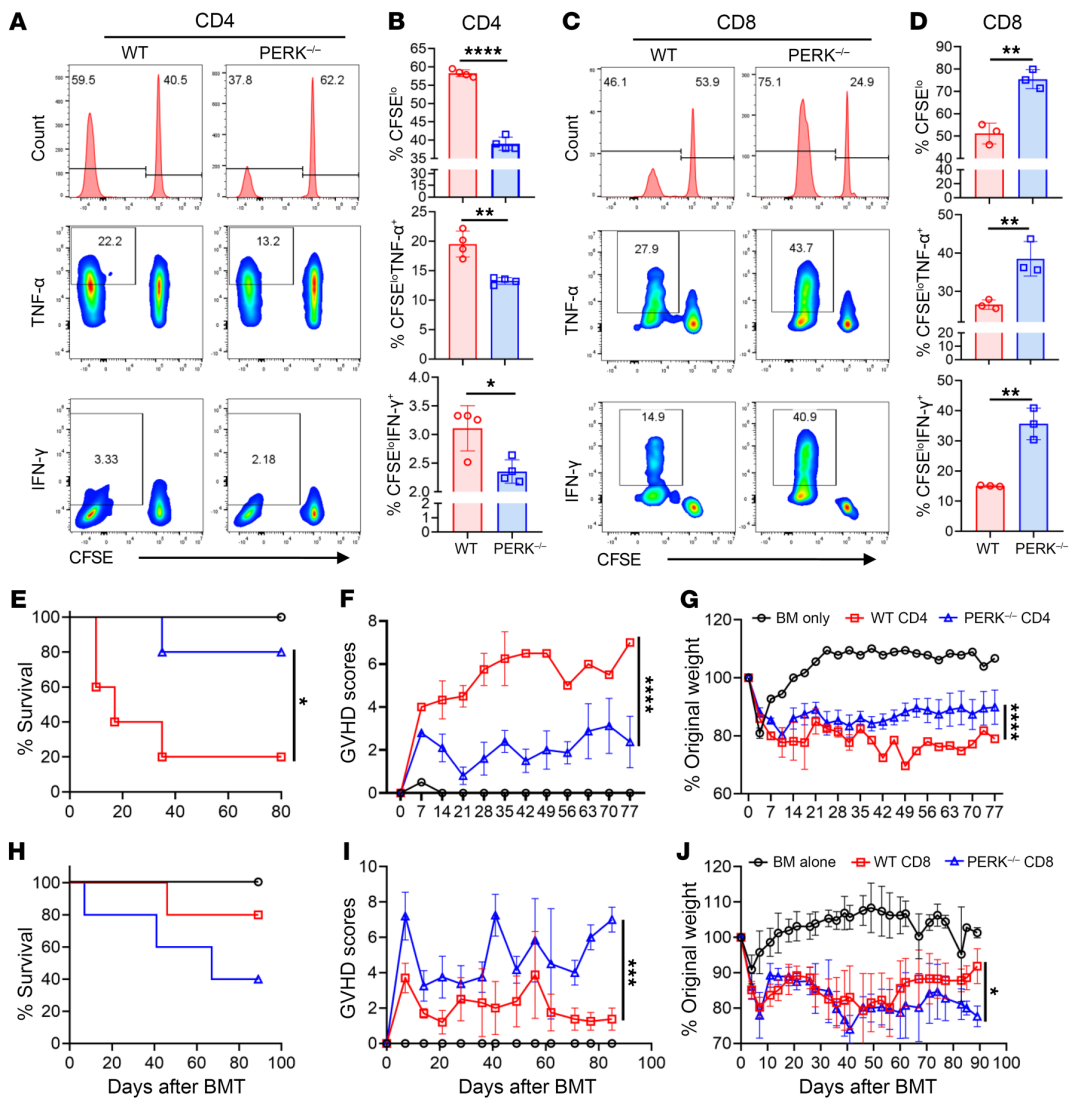
PERK differentially regulates CD4^+^ and CD8^+^ T cell responses to alloantigens. (**A** and **B**) CD4^+^ T cells isolated from WT or PERK-cKO mice were stimulated with allogeneic APCs from BDF1 mice for 4 days; proliferation (CFSE^lo^) of CD4^+^ T cells and levels of proinflammatory cytokines (TNF-α, IFN-γ) in CD4^+^ T cells were analyzed using flow cytometry (**A**). Percentages of CFSE^lo^CD4^+^, CFSE^lo^TNF-α^+^CD4^+^, and CFSE^lo^IFN-γ^+^CD4^+^ T cells among gated H2K^d–^CD4^+^ T cells are shown (**B**). (**C** and **D**) CD8^+^ T cells isolated from WT or PERK-cKO mice were stimulated with allogeneic APCs from BDF1 mice for 4 days; proliferation (CFSE^lo^) of CD8^+^ T cells and levels of proinflammatory cytokines (TNF-α, IFN-γ) in CD8^+^ T cells were analyzed by flow cytometry (**C**). Percentages of CFSE^lo^CD8^+^, CFSE^lo^TNF-α^+^CD8^+^, and CFSE^lo^IFN-γ^+^CD8^+^ T cells among gated H2K^d–^CD8^+^ T cells are shown (**D**). (**E**–**G**) Lethally irradiated BALB/c recipients were injected with TCD-BM cells (5 × 10^6^) alone or together with CD4^+^ T cells (1.25 × 10^6^) from WT B6 or PERK-cKO donors. Survival (**E**), GVHD scores (**F**), and body weight (**G**) of BALB/c recipients were monitored through 80 days after BMT; *n* = 5 per BMT group. (**H**–**J**) Lethally irradiated BALB/c recipients (900 cGy) were injected with TCD-BM cells (5 × 10^6^) alone or along with CD8^+^ T cells (2.5 × 10^6^) from WT B6 or PERK-cKO donors and CD25-removed CD4^+^ T cells (0.5 × 10^6^) from WT B6 mice. Survival (**H**), GVHD scores (**I**), and body weight (**J**) of BALB/c recipients were monitored through 80 days after BMT; *n* = 5 per BMT group. Log-rank (Mantel-Cox) test (**E** and **H**) and non-parametric Mann-Whitney *U* test (**F**, **G**, **I**, and **J**) were used to compare groups. Data in **B** and **D** are represented as mean ± SD with biological replicates; significance was determined using a 2-tailed unpaired Student’s *t* test. *P < 0.05, **P < 0.01, ***P < 0.001, ****P < 0.0001.

To explore how PERK regulates T cell allogeneic responses, CD4^+^ or CD8^+^ T cells were isolated from WT or PERK-cKO mice and stimulated separately with allogeneic APCs from BDF1 mice in vitro. We found that PERK deficiency reduced the proliferation and cytokine secretion of CD4^+^ T cells after allogeneic stimulation (Figure 4, A and B). In contrast, PERK deficiency promoted the proliferation and cytokine secretion of CD8^+^ T cells (Figure 4, C and D). These results suggest that PERK differentially regulates the allogeneic responses of CD4^+^ and CD8^+^ T cells in vitro. To determine whether PERK would regulate CD4^+^ and CD8^+^ T cell alloresponses in vivo, CD4^+^ or CD8^+^ T cells were isolated from WT or PERK-cKO mice and transferred into lethally irradiated BALB/c mice separately. PERK-deficient CD4^+^ T cells showed reduced ability to induce GVHD (Figure 4, E–G). In sharp contrast, PERK-deficient CD8^+^ T cells had increased ability to cause GVHD (Figure 4, H–J). Consistent with GVHD pathogenicity, PERK-deficient CD8^+^ T cells were found to produce higher levels of IFN-γ, TNF-α, and GM-CSF in allogeneic recipients (Supplemental Figure 9, A and B). Taken together, these results suggest that PERK distinctly regulates CD4^+^ and CD8^+^ T cell allogeneic responses.

To further investigate whether the effects of AMG44 on T cell allogeneic responses depend on PERK expression of T cells, we compared the responses of PERK-deficient and WT T cells following AMG44 treatment. We found that AMG44 treatment significantly inhibited the proliferation or cytokine production of WT CD4^+^ T cells after allostimulation but not PERK-cKO CD4^+^ T cells (Supplemental Figure 10, A and B), suggesting the specificity of AMG44 in the inhibition of PERK. AMG44 treatment did not significantly reduce the proliferation of and cytokines in PERK-deficient CD8^+^ T cells as compared with vehicle-treated PERK-deficient CD8^+^ T cells (Supplemental Figure 10, C and D). To evaluate the specificity of AMG44 in vivo, we transplanted WT or PERK-cKO CD8^+^ T cells into allogeneic recipients and treated them with AMG44. AMG44 treatment promoted GVHD induced by WT CD8^+^ T cells (Supplemental Figure 10, E and F), which mimics genetic PERK deficiency (Figure 4, H–J). However, AMG44 treatment ameliorated GVHD severity induced by PERK-deficient CD8^+^ T cells (Supplemental Figure 10, E and F), suggesting that PERK inhibition could suppress CD4 helper function or non-T cells such as APCs.

### PERK inhibits CD8^+^ T cell responses.

**Figure 5:**
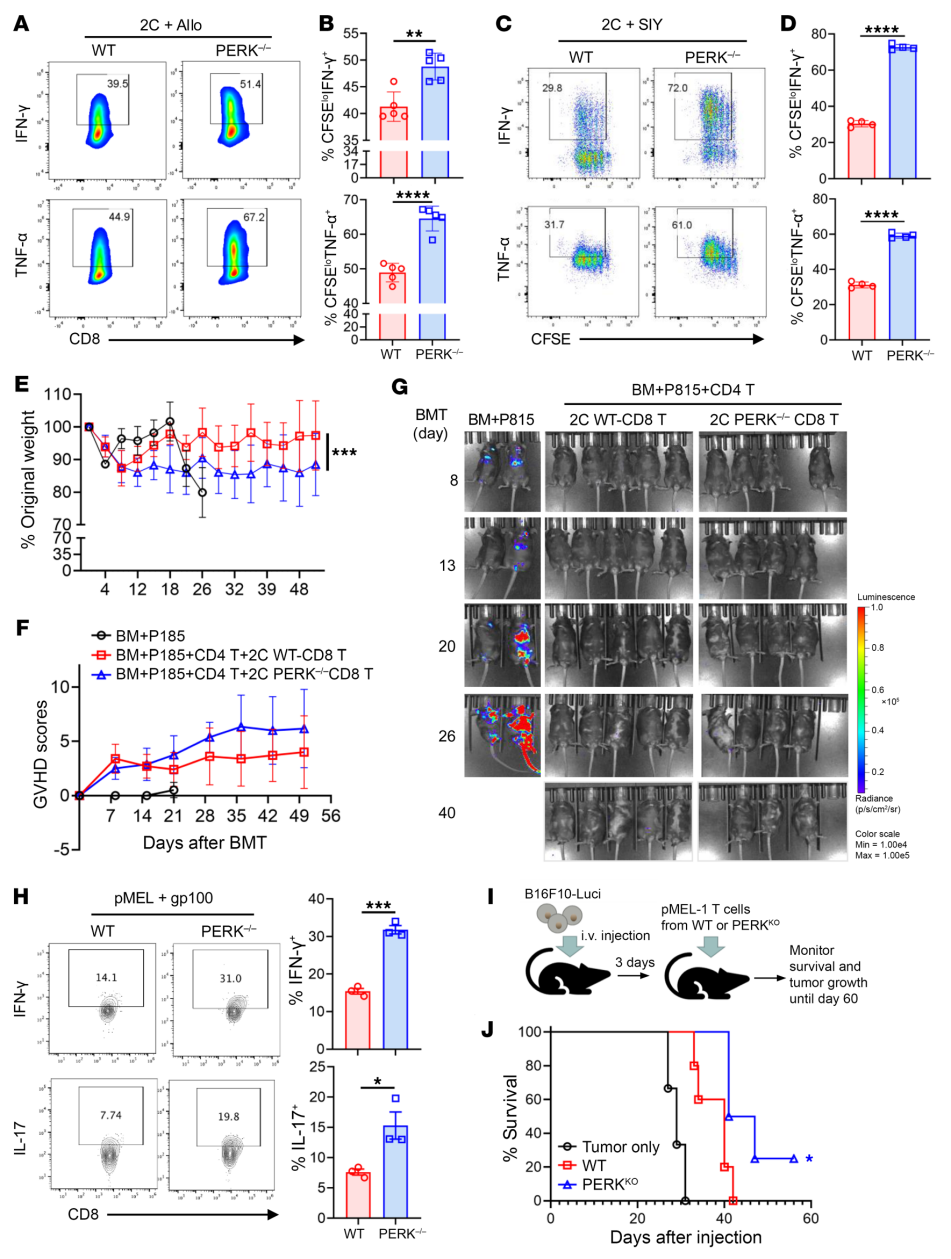
PERK inhibits CD8^+^ T cell responses. (**A** and **B**) CD8^+^ T cells isolated from 2C transgenic WT or PERK-cKO mice were labeled with CFSE and stimulated with allogeneic APCs from BDF1 mice for 4 days; levels of proinflammatory cytokines (IFN-γ, TNF-α) in CD8^+^ T cells were analyzed by flow cytometry (**A**). Percentages of CFSE^lo^IFN-γ^+^CD8^+^ and CFSE^lo^TNF-α^+^CD8^+^ T cells among gated H2K^d–^CD8^+^ T cells are shown (**B**). (**C** and **D**) CD8^+^ T cells isolated from 2C transgenic WT or PERK-cKO mice were labeled with CFSE and stimulated with SIY peptides (10 nM) and syngeneic B6 APCs (Ly5.1); levels of cytokines were analyzed similarly. (**E**–**G**) Lethally irradiated BDF1 mice (1,200 cGy) received TCD-BM cells (5 × 10^6^) alone or along with P815-luc cells (5,000) with or without CD25-removed T cells (3 × 10^6^) from 2C WT or PERK-cKO donors. Body weight loss (**E**) and GVHD scores (**F**) were monitored after BMT. Tumor growth in BDF1 recipients was monitored via BLI (**G**); *n* = 5 per BMT group. (**H**) CD8^+^ T cells from pMEL WT or PERK-cKO mice were stimulated with gp100 peptides and B6 APCs (Ly5.1) for 3 days; levels of IFN-γ and IL-17 in CD8^+^ T cells were analyzed. Percentages of IFN-γ^+^CD8^+^ and IL-17^+^CD8^+^ T cells among gated CD45.1^–^CD8^+^ T cells are shown. (**I** and **J**) Lethally irradiated BDF1 mice received TCD-BM cells (5 × 10^6^) along with B16F10-luc cells with or without CD25-removed T cells (3 × 10^6^) from pMEL WT or PERK-cKO donors; *n* = 3 for tumor only, *n* = 5–6 for WT or PERK-cKO (**I**). Survival (**J**) was monitored after BMT. Log-rank (Mantel-Cox) test (**J**) and non-parametric Mann-Whitney *U* test (**E** and **F**) were used to compare groups. Data in **B**, **D**, and **H** are represented as mean ± SD with biological replicates; significance was determined using a 2-tailed unpaired Student’s *t* test. *P < 0.05, **P < 0.01, ***P < 0.001, ****P < 0.0001.

We further investigated how PERK regulates CD8^+^ T cell responses, which may contribute to the preserved GVL effect seen when PERK was deficient or inhibited. CD8^+^ T cells were purified from 2C T cell receptor–transgenic (TCR-Tg) WT or PERK-cKO mice and stimulated with allogeneic APCs or SIYRYYGL (SIY) peptide. As compared with WT 2C T cells, PERK-deficient 2C T cells showed increased activation, reflected by higher IFN-γ and TNF-α production, in response to alloantigens (Figure 5, A and B) as well as peptide antigen in vitro (Figure 5, C and D). These results suggest that PERK restricts CD8^+^ T cell responses in vitro. To further investigate the role of PERK in donor CD8^+^ T cell–mediated GVH/GVL responses in vivo, CD8^+^ T cells were isolated from 2C TCR-Tg WT or PERK-cKO mice and transferred into lethally irradiated BDF1 mice. To facilitate CD8^+^ T cell response in vivo, a small number of CD4^+^ T cells from normal B6 mice were cotransferred with 2C T cells, as CD8^+^ 2C T cells alone can only induce mild GVHD limited to the hematopoietic compartment (33). We then observed that PERK-deficient 2C T cells were trending toward higher pathogenesis of GVHD (Figure 5, E and F) while maintaining a strong GVL effect (Figure 5G). To investigate the role of PERK more specifically in antitumor response of CD8^+^ T cells, we stimulated pMEL TCR-Tg cells with specific antigen gp100 in vitro and found that PERK-deficient CD8^+^ T cells secreted higher levels of IFN-γ and IL-17 (Figure 5H). Furthermore, pMEL TCR-Tg PERK-deficient CD8^+^ T cells prolonged the survival of mice bearing B16F10 melanoma (Figure 5, I and J). Taken together, these results indicate that PERK deficiency promotes CD8^+^ T cell responses.

### PERK distinctly regulates T cell responses to allogeneic versus polyclonal stimulation.

To explore potential mechanisms by which PERK regulates CD4^+^ and CD8^+^ T cell responses, we examined the role of PERK in T cell responses after stimulation with allogeneic APCs or anti-CD3/CD28. In gated CD4^+^ T cells, PERK deficiency reduced the production of IFN-γ and GM-CSF upon allogeneic stimulation, but increased cytokine production upon polyclonal stimulation (Supplemental Figure 11, A–C). In gated CD8^+^ T cells, PERK deficiency had little impact on cytokine production upon allogeneic stimulation, but increased cytokine production upon polyclonal stimulation (data not shown). Thus, PERK appeared to distinctly regulate T cell allogeneic and polyclonal responses, especially CD4^+^ T cells. Cao et al. reported that PERK deficiency promotes T cell activation and antitumor response by decreasing *Ddit3* mRNA (encoding CHOP) and CHOP protein while increasing T-bet expression (20). To determine how PERK differentially regulates T cell response to polyclonal versus allogeneic stimulation, we measured Ddit3, CHOP, and T-bet levels after T cell stimulation. Consistent with the observations by Cao et al., we found that PERK-deficient T cells, both CD4^+^ and CD8^+^, decreased Ddit3 and CHOP expression while increasing T-bet expression after polyclonal stimulation (Supplemental Figure 11, D–G). In sharp contrast, PERK-deficient T cells increased Ddit3 and CHOP expression while decreasing T-bet expression after allogeneic stimulation, especially in CD4^+^ T cells (Supplemental Figure 11, D–G).

To investigate how PERK regulates T cell response in polyclonal versus allogeneic stimulation at the metabolic level, we found that mitochondrial content was significantly reduced in PERK-deficient CD4^+^ but not CD8^+^ T cells after allogeneic stimulation (Supplemental Figure 11, H and I). In contrast, mitochondrial content was significantly increased in PERK-deficient CD4^+^ and CD8^+^ T cells after polyclonal stimulation (Supplemental Figure 11, H and I). We then measured T cell metabolism by Seahorse analysis. In the absence of PERK, T cells reduced oxidative phosphorylation, as reflected by oxygen consumption rate (OCR), upon allogeneic stimulation, whereas T cells had a similar level of oxidative phosphorylation upon polyclonal stimulation regardless of PERK expression (Supplemental Figure 11, J and K). Similarly, PERK-deficient T cells significantly reduced glycolysis reflected by extracellular acidification rate (ECAR) upon allogeneic stimulation, whereas these T cells significantly increased glycolysis upon polyclonal stimulation (Supplemental Figure 11, L and M). These results suggest that PERK differentially regulates T cell oxidative phosphorylation and glycolysis upon polyclonal versus allogeneic stimulation.

### PERK regulates T cell allogeneic responses through ERAD signaling.

**Figure 6:**
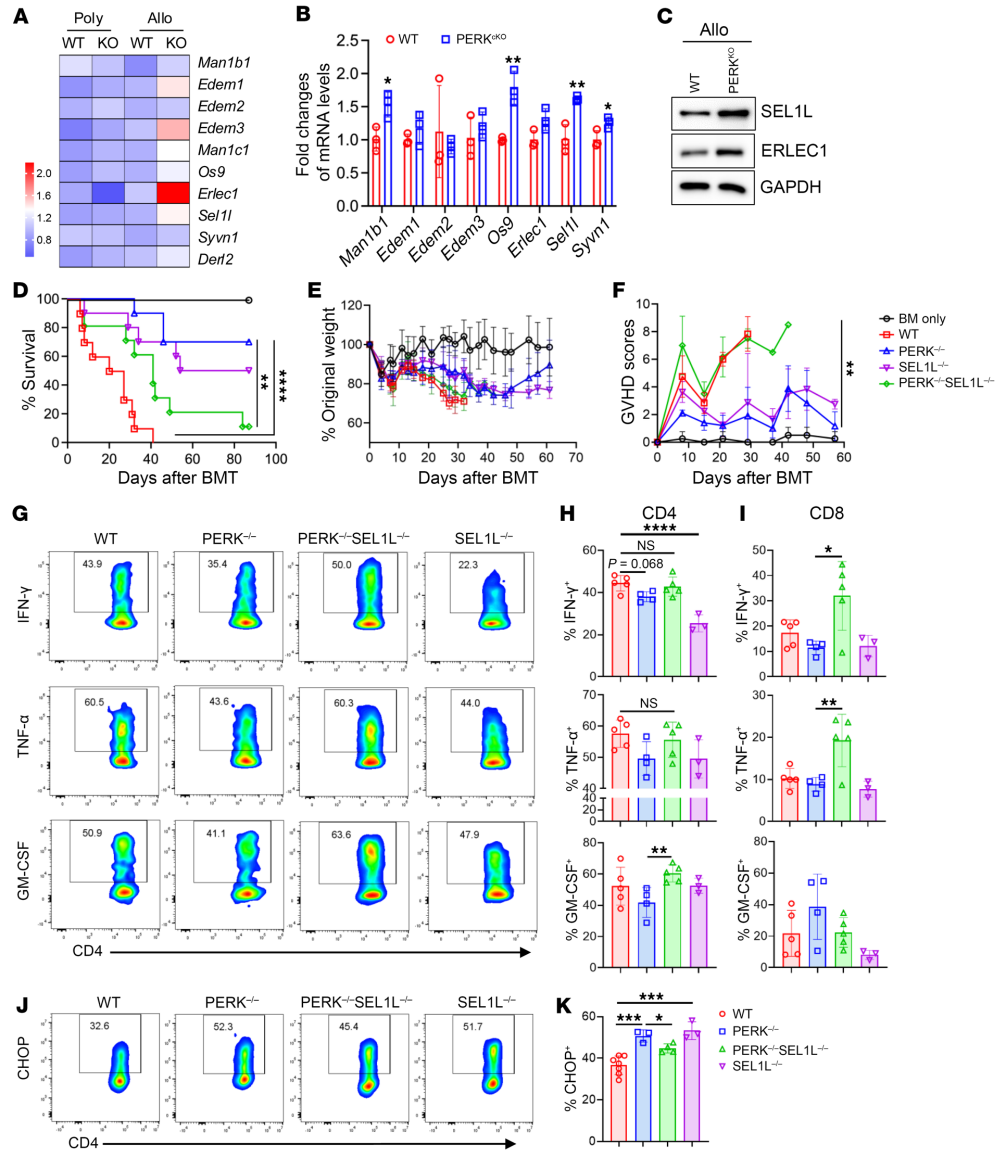
PERK regulates T cell allogeneic responses through ERAD. (**A**) T cells from WT or PERK-cKO mice stimulated with anti-CD3/CD28 (2 μg/mL) or allogeneic APCs for 4 days were performed RNA-Seq. Differentially expressed ERAD-associated genes are shown. (**B** and **C**) T cells from WT or PERK-cKO mice were stimulated by allogeneic APCs; mRNA levels of indicated were analyzed by quantitative real-time PCR (**B**). (**C**) Protein levels of SEL1L, ERLEC1, and GAPDH were evaluated by Western blot. (**D**–**F**) Lethally irradiated BALB/c mice received TCD-BM cells (5 × 10^6^) alone or together with T cells (1.25 × 10^6^) from WT, PERK-cKO, SEL1L-cKO, or PERK/SEL1L-double-KO (dKO) donors. Survival (**D**), body weight (**E**), and GVHD scores (**F**) of BALB/c recipients were monitored after BMT; *n* = 9–10 combined from 2 replicate experiments. (**G**–**K**) Lethally irradiated BALB/c mice received BM cells (5 × 10^6^) isolated from Rag1-KO mice along with T cells (1.25 × 10^6^) purified from WT, PERK-cKO, SEL1L-cKO, or PERK/SEL1L-dKO donors. (**G**) Levels of IFN-γ, TNF-α, and GM-CSF in donor CD4^+^ or CD8^+^ T cells of livers from the recipients were analyzed. (**H**) Percentages of IFN-γ^+^CD4^+^, TNF-α^+^CD4^+^, and GM-CSF^+^CD4^+^ T cells among gated H2K^b+^CD4^+^ T cells are shown. (**I**) Percentages of IFN-γ^+^CD8^+^, TNF-α^+^CD8^+^, and GM-CSF^+^CD8^+^ T cells among gated H2K^b+^CD8^+^ T cells are shown. (**J**) Levels of CHOP in donor CD4^+^ T cells were analyzed. (**K**) Mean fluorescence intensity of CHOP among gated H2K^b+^CD4^+^ T cells is displayed. Log-rank (Mantel-Cox) test (**D**) and non-parametric Mann-Whitney *U* test (**E** and **F**) were used to compare groups. Data in **B** are represented as mean ± SD; significance was determined using a 2-tailed unpaired Student’s *t* test. Data in **H**, **I**, and **K** are represented as mean ± SD; significance was determined using a 1-way ANOVA test. *P < 0.05, **P < 0.01, ***P < 0.001, ****P < 0.0001.

To further elucidate the mechanism of PERK differentially regulating T cell allogeneic and antitumor responses, we performed transcriptomics analyses via bulk RNA-Seq on WT and PERK-deficient T cells after allogeneic and polyclonal stimulation. All the differentially expressed genes in PERK-deficient versus WT T cells after allogeneic and polyclonal stimulation are presented in volcano plots (Supplemental Figure 12). The transcriptomics data showed that genes associated with ER-associated protein degradation (ERAD), including Sel1l and Erlec1, were significantly increased in PERK-cKO compared with WT T cells upon allogeneic but not polyclonal stimulation (Figure 6A). Increased mRNA levels of ERAD-associated genes were confirmed with quantitative PCR in PERK-cKO T cells upon allogeneic stimulation (Figure 6B). Consistently, protein levels of SEL1L and ERLEC1 were also increased in allogeneic PERK-deficient T cells (Figure 6C). When stimulated with alloantigen separately, PERK-deficient CD4^+^ and CD8^+^ T cells upregulated SEL1L (Supplemental Figure 13A) and thus decreased amyloid β-protein aggregation (Supplemental Figure 13, B and C). However, SEL1L protein levels were decreased in PERK-deficient CD8^+^ T cells after polyclonal stimulation (Supplemental Figure 13D) with increased accumulation of aggregates (Supplemental Figure 13E). In addition, protein levels of CHOP were increased in PERK-deficient CD4^+^ T cells but not CD8^+^ T cells after allogeneic stimulation (Supplemental Figure 13F). In contrast, CHOP protein levels were markedly decreased in PERK-deficient CD8^+^ T cells after polyclonal stimulation (Supplemental Figure 13G), which is consistent with reported data (20). To determine whether PERK regulates T cell allogeneic responses through regulating the ERAD pathway, we stimulated T cells with allogeneic APCs or anti-CD3/CD28 in the presence of kifunensine, an ERAD inhibitor (34). Kifunensine reversed the defect in IFN-γ production in PERK-deficient CD4^+^ T cells upon allogeneic stimulation (Supplemental Figure 14, A and B), but not upon polyclonal stimulation (data not shown). Furthermore, kifunensine also reversed the reduction of mitochondria components and elevation of CHOP levels in PERK-deficient CD4^+^ T cells after alloantigen stimulation (Supplemental Figure 14, C and D). These data support that the elevated ERAD contributes to the impaired allogeneic T cell responses in the absence of PERK.

Because the SEL1L/HRD1 axis plays a central role in the ERAD pathway (35), we determined to test whether PERK interacts with SEL1L. Indeed, we observed that PERK binds to SEL1L in an activated human T cell line (Supplemental Figure 15A). To verify whether PERK’s regulation of T cell allogeneic responses is dependent on the SEL1L-mediated ERAD pathway, we generated SEL1L/PERK-double-knockout mice, and demonstrated that additional SEL1L deficiency reversed the defects of PERK-cKO CD4^+^ T cells in response to alloantigen, as reflected by mitochondrial components, proliferation, and TNF-α production in vitro (Supplemental Figure 15, B–E). Moreover, SEL1L deficiency largely reversed the reduction of the ability of PERK-cKO T cells in the induction of GVHD (Figure 6, D–F). Consistent with in vitro studies, we found that PERK cKO did not reduce cytokine production (Figure 6, G–I) or increase CHOP expression (Figure 6, J and K) when SEL1L was absent as well. These results elucidate that PERK regulates T cell allogeneic responses and GVHD induction through the SEL1L-mediated ERAD pathway.

### Pharmacological inhibition of PERK reduces GVHD induced by human T cells while increasing GVL activity.

**Figure 7:**
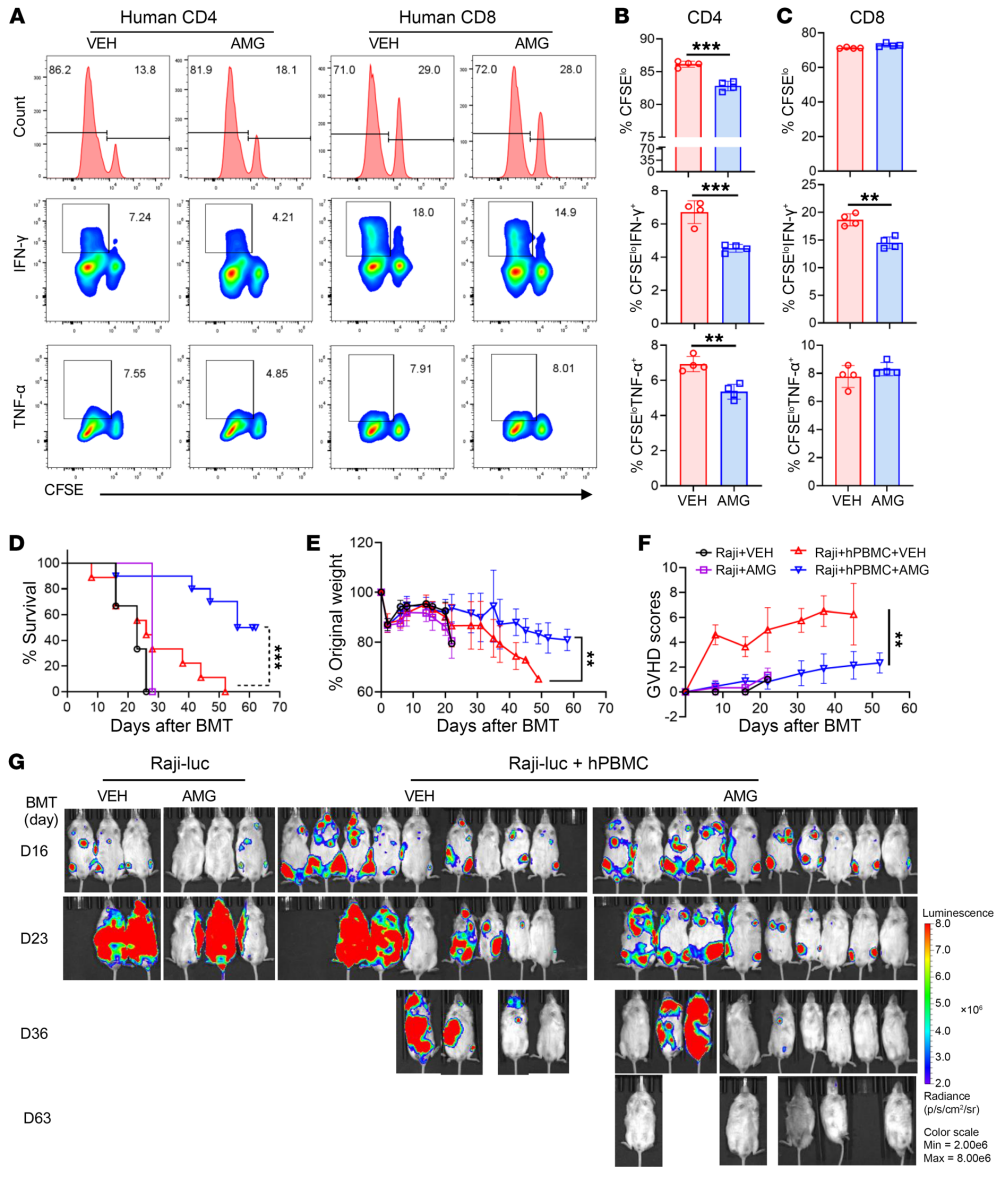
Inhibition of PERK reduces GVHD induced by human T cells while maintaining GVL activity. (**A**–**C**) T cells were isolated from human HLA-A2^–^ PBMCs, labeled with CFSE, and stimulated with allogeneic HLA-A2^+^ APCs (TCD-PBMCs); proliferation and production of IFN-γ, TNF-α, and granzyme B (GZMB) of donor CD4^+^ and CD8^+^ T cells were analyzed by flow cytometry (**A**). Percentages of CFSE^lo^CD4^+^, IFN-γ^+^CD4^+^, TNF-α^+^CD4^+^, and GZMB^+^CD4^+^ T cells among gated HLA-A2^–^CD4^+^ T cells are shown (**B**). Percentages of CFSE^lo^CD8^+^, IFN-γ^+^CD8^+^, TNF-α^+^CD8^+^, and GZMB^+^CD8^+^ T cells among gated HLA-A2^–^CD8^+^ T cells are shown (**C**). (**D**–**G**) Sublethally irradiated NSG mice were injected with Raji-luc cells along with or without human PBMCs (8 × 10^6^). Recipient mice were treated with vehicle (VEH) or AMG44 (5 mg/kg) every other day from day 0 to day 14. Survival (**D**), body weight loss (**E**), and GVHD scores (**F**) were monitored through 60 days after BMT; *n* = 3 for Raji with VEH or AMG, *n* = 9–10 for Raji and PBMCs with VEH or AMG combined from 2 replicate experiments. Tumor growth in NSG recipients was monitored via BLI (**G**). Log-rank (Mantel-Cox) test (**D**) and non-parametric Mann-Whitney *U* test (**E** and **F**) were used to compare groups. Data in **B** and **C** are represented as mean ± SD with biological replicates; significance was determined using a 2-tailed unpaired Student’s *t* test. **P < 0.01, ***P < 0.001.

To promote translation, we next investigated the role of PERK in human T cell responses. T cells were isolated from human PBMCs, labeled with CFSE, and stimulated with allogeneic APCs (T cell–depleted [TCD] PBMCs) with or without AMG44. We observed that AMG44 supplement suppressed proliferation and reduced IFN-γ and TNF-α production by CD4^+^ T cells in response to allogeneic stimulation, but did not impact proliferation and TNF-α production of CD8^+^ T cells, while slightly decreasing IFN-γ production (Figure 7, A–C). Consistent with mouse data, AMG44 promoted IFN-γ and TNF-α in CD4^+^ and CD8^+^ T cells stimulated with anti-CD3/CD28 (Supplemental Figure 16, A–C). We then conducted an in vivo study using a xenograft GVHD model and found that AMG44 treatment reduced GVHD induced by human T cells, as reflected by better GVHD survival (Figure 7D) and reduced body weight loss (Figure 7E) and clinical scores (Figure 7F). More importantly, AMG44 also preserved GVL activity (Figure 7G). These results indicate that PERK inhibition can preserve GVL effects while attenuating GVHD induced by human T cells.

## Discussion

The present study demonstrates that targeting the ER stress sensor PERK suppresses GVHD while preserving GVL activity. PERK promotes differentiation of CD4^+^ T cells toward Th1 and Th17 subsets while limiting their differentiation into the Treg subset. On the other hand, PERK negatively regulates CD8^+^ T cell–mediated allogeneic responses and antitumor responses. Thus, PERK differentially regulates CD4^+^ and CD8^+^ T cell allogeneic responses. This study provides evidence that PERK is a promising ER stress target to reduce GVHD while maintaining GVL effect.

Our findings reveal that PERK promotes the proliferation of, and proinflammatory cytokines in, allogeneic CD4^+^ T cells, thereby exacerbating GVHD. On the other hand, PERK inhibits the proliferation of, and proinflammatory cytokines in, allogeneic CD8^+^ T cells that may contribute to the preserved GVL effect. When total T cells were transferred into allogeneic recipients, PERK-cKO T cells significantly reduced their ability to induce GVHD while preserving the GVL effect (Figures 1–3 and 6). Similarly, treatment with PERK inhibitor significantly reduced GVHD while sparing GVL activity (Figures 3 and 7). Under the condition in which CD8^+^ T cells deficient in PERK increased allogeneic response, CD4 helper was provided by WT CD4^+^ T cells (Figures 4 and 5). We interpret that, when PERK is deficient or blocked, CD4^+^ T cells are incapable of providing a strong helper to CD8^+^ T cells, and therefore CD8^+^ T cells even lacking PERK cannot mount a powerful allogeneic response to induce severe GVHD while mediating an adequate GVL response. Taking these results together, we reason that inhibition of PERK can be translated to the clinic for controlling GVHD and leukemia relapse after allogeneic hematopoietic cell transplantation.

The activating effects of PERK on CD4^+^ T cells in allogeneic responses are consistent throughout the study. However, the effect of PERK on CD8^+^ T cells appears to be somewhat inconsistent, and we interpret the data in several ways as the following: (a) When total T cells were stimulated with alloantigen, the degree of CD4 helper influenced CD8 responses. Under this condition, we observed that, in the absence of PERK on both types of T cells, CD8^+^ T cell response was not increased (Figure 2, C and D, Supplemental Figure 4, C–E, and Supplemental Figure 11, G and I). However, when purified CD8^+^ T cells were stimulated alone without CD4 helper in vitro, the suppressive effects of PERK on CD8^+^ T cells became apparent (Figure 4, A–D, and Figure 5, A–D). Under in vivo conditions, the suppressive effects of PERK on CD8^+^ T cells were also obvious (Figure 4, F–J, Figure 5, E–G, and Supplemental Figure 10, C and D), which was likely due to a strong helper provided by additional WT CD4^+^ T cells. (b) With regard to human T cells, CD8^+^ T cell response was slightly decreased when PERK was inhibited by AMG44 (Figure 7, A–C). It is likely that impaired CD4 helper function impacted CD8^+^ T cell response. The interpretation is supported by the observation that the response of human CD8^+^ T cells was significantly increased when T cells were activated by strong polyclonal stimulation in vitro (Supplemental Figure 16). One other possibility is that, unlike T cell–specific PERK deficiency (Figures 4 and 5), the PERK inhibition could impact APCs as well (36). (c) Most importantly, pharmacological inhibition of PERK significantly reduced GVHD while preserving the GVL effect in vivo regardless of experimental models, which set a strong rationale for clinical translation.

CHOP as a PERK downstream target was reported to impair the effector function of tumor-infiltrating T cells in murine tumor models. In ovarian cancer patients, increased expression of CHOP in tumor-infiltrating CD8^+^ T cells was associated with poor clinical outcome (20). In the current study, when CD4^+^ or CD8^+^ T cells were stimulated separately, we found that PERK deficiency increased CHOP levels in CD4^+^ T cells but not in CD8^+^ T cells after allogeneic stimulation (Supplemental Figure 13F). CHOP levels were decreased in PERK-deficient CD8^+^ T cells upon polyclonal stimulation (Supplemental Figure 13G). The finding that PERK distinctly regulates CD4^+^ and CD8^+^ T cell allogeneic response was further supported by in vivo studies (Figures 4 and 5). However, we observed that PERK deficiency increased CHOP levels in CD8^+^ T cells when CD4^+^ and CD8^+^ T cells were stimulated with alloantigen together in vitro (Supplemental Figure 8, D and E). We interpret that the upregulation of CHOP in PERK-deficient CD8^+^ T cells was impacted by reduced helper from PERK-deficient CD4^+^ T cells in the mixed culture. CHOP was reported to directly repress the expression of T-bet in CD8^+^ T cells (20). Consistently, we found that PERK suppressed T-bet levels in CD8^+^ and CD4^+^ T cells after polyclonal stimulation (Supplemental Figure 8, F and G). Thus, distinct regulation of CD4^+^ versus CD8^+^ T cell allogeneic response by PERK may occur at least partially through its control of CHOP expression levels.

Abrogating PERK activity in T cells was shown to result in decreased mitochondrial reactive oxygen species and enhanced antitumor immunity in vivo (37). Additionally, PERK was reported to regulate mitochondrial biology during ER stress (38). Whether PERK affects the metabolism of allogeneic T cells or how it affects them is essentially uncharacterized. In our study, we found that PERK deletion reduces the maximal respiration and glycolysis in allogeneic T cells while promoting glycolysis in T cells after polyclonal stimulation, indicating that PERK differentially regulates the oxidative phosphorylation and glycolysis of allogeneic T cells and antitumor T cells (Supplemental Figure 11, J–M).

The SEL1L/HRD1 axis is a key component of the ERAD signaling pathway (35). SEL1L/HRD1 ERAD was reported to control the identity of β cells via TGF-β signaling (39). SEL1L deficiency in T cells promotes Th1/Th17 differentiation and exacerbates EAE (22). Targeting SEL1L significantly promotes the allogeneic responses of T cells in vitro (data not shown), but SEL1L deficiency in donor T cells did not exacerbate GVHD in vivo (Figure 6, D–F). SEL1L was reported to regulate the survival and homeostasis of CD8^+^ T cells through the PERK signaling pathway (23). In a GVHD model, we demonstrated that PERK interacts with and regulates SEL1L, and additional SEL1L deletion reversed the defects of PERK-cKO T cell allogeneic response (Figure 6, D–F). Thus, we interpret that PERK impacts T cell allogeneic responses and GVHD induction at least partially through the ERAD pathway. In humans, SEL1L ERAD has been reported to be involved in children’s neurodevelopmental disorders, such as developmental delay, intellectual disability, microcephaly, facial dysmorphisms, hypotonia, and/or ataxia (40). The role of SEL1L in human GVHD needs to be further investigated.

Targeting ER stress by using TUDCA can reduce GVHD while preserving GVL activity. Interestingly, TUDCA still reduced GVHD while preserving GVL activity even when XBP1 was absent in donor T cells (Supplemental Figure 8, E–G). Thus, PERK, not XBP1, is a potential target for TUDCA to alleviate GVHD and preserve GVL effect. The metabolite trimethylamine *N*-oxide induced pyroptosis in tumor cells by activating the ER stress kinase PERK and thus enhanced CD8^+^ T cell–mediated antitumor immunity in triple-negative breast cancer (41). AMG44 was reported to be a more specific inhibitor that targets PERK compared with other PERK inhibitors, such as GSK2606414 and GSK2656157 (19). In a murine GVHD model, we found that AMG44 treatment significantly reduced GVHD while preserving GVL activity (Figure 3, E–H). Pharmacologically targeting PERK with AMG44 also reduced human T cell–mediated GVHD while preserving GVL activity (Figure 7, D–G). These findings proved that PERK is a promising target for alleviating GVHD while preserving the GVL effect, supporting clinical investigation to validate targeting of PERK as a potential therapeutic strategy in the control of GVHD and leukemia relapse.

## Methods

### Sex as a biological variable.

Our study examined male and female animals, and similar findings are reported for both sexes.

### Mice and human T cells.

C57BL/6 (B6, H2K^b^, Ly5.2^+^ or B6, H2K^b^, Ly5.1^+^), BALB/c (H2K^d^), and (B6 × DBA2)F1 (B6D2F1, H2K^b/d^) mice were purchased from the National Cancer Institute Mouse Repository (Frederick, Maryland, USA). NOD/SCID/IL2Rg^–/–^ (NSG; HLA-A2^+^) and PERK^fl/fl^ mice were purchased from The Jackson Laboratory. The XBP1^fl/fl^ and SEL1L^fl/fl^ strains were provided by Chih-Chi Andrew Hu (42) and Ling Qi (43), respectively. Mice with conditional knockout (cKO) of PERK, XBP1, or SEL1L on B6 background were produced by cross-breeding of PERK^fl/fl^, XBP1^fl/fl^, and SEL1L^fl/fl^ mice with CD4-Cre–expressing mice, respectively. PERK/SEL1L-double-knockout (dKO) strain was produced by cross-breeding of PERK^fl/fl^ and SEL1L^fl/fl^ mice with CD4-Cre^+^ mice. B6 2C TCR-transgenic (Tg) and PERK-cKO mice were bred by crossing of 2C Tg with CD4-Cre^+^ or PERK-cKO mice. B6 pMEL Tg and PERK-cKO mice were bred by crossing of pMEL Tg mice with CD4-Cre^+^ or PERK-cKO mice. Human T cells were isolated from the peripheral blood mononuclear cells (PBMCs) of healthy donors, purchased from Research Blood Components LLC.

### Cell culture and reagents.

Luciferase-overexpressing P815 (P815-luc) and MLL-AF9-GFP cells were given by Pavan Reddy and Sophie Paczesny at the University of Michigan and Indiana University, respectively. Luciferase-overexpressing B16F10 (B16F10-luc) cells were purchased from IVIS Imaging Co. Jurkat E6.1 cells were purchased from ATCC. Luciferase-overexpressing Raji (Raji-luc) cells were given by Defu Zeng at the City of Hope Medical Center (Los Angeles, California). P815-luc, MLL-AF9-GFP, B16F10-luc, Jurkat E6.1, and Raji-luc cell lines as well as all mouse and human primary T cells were cultured in RPMI 1640 medium purchased from Gibco supplemented with 10% FBS (Gibco), as well as penicillin/streptomycin (Corning), nonessential amino acids (Corning), sodium pyruvate (Corning), and 2-mercaptoethanol (Gibco).

AMG44 was purchased from Tocris Bioscience. Tauroursodeoxycholic acid (TUDCA), kifunensine, and the small-molecule Nrf2 activator sulforaphane (SFN) were purchased from Cayman Chemical. MitoTracker Green and LIVE-DEAD Yellow were purchased from Invitrogen.

### Induction and assessment of GVHD.

BALB/c recipients were exposed to 900 cGy total-body irradiation (TBI) using cesium-137 irradiator before bone marrow transplantation (BMT), and then intravenously given T cell–depleted BM (TCD-BM) cells (5 × 10^6^ per mouse) from C57BL/6 (B6) or RAG1-KO donors with or without T cells (1.25 × 10^6^) purified from B6 donors. The recipients were monitored through body weight loss and GVHD scores twice a week. (B6 × DBA2)F1 (BDF1) recipients were exposed to 1,200 cGy TBI with 2 split doses before BMT, and then intravenously given TCD-BM cells (5 × 10^6^) from B6 or RAG1-KO donors with or without mastocytoma P815-luc cells (5,000), along with or without CD25-depleted T cells (3 × 10^6^) purified from spleen and lymph node cells of B6 donors. For xenograft BMT model, NSG mice were exposed to 250 cGy TBI before BMT, and then intravenously given Raji-luc cells (1 × 10^6^), along with or without HLA-A2^–^ human PBMCs (8 × 10^6^). The recipients were monitored through body weight loss and GVHD scores twice a week. Tumor growth was monitored using IVIS Spectrum CT (PerkinElmer). Tumor and GVHD mortality was distinguished by bioluminescent imaging signal intensity and GVHD manifestation. Detailed information on T cell isolation and TCD-BM cells has been described previously (44, 45).

### Antibodies and flow cytometry.

Monoclonal antibodies (mAbs) specific for mouse H2K^b^ (clone AF6-88.5), CD45.1 (clone A20), CD45.2 (clone 104), CD4 (clone RM4-5), CD8 (clone 53-6.7), B220 (clone RA3-6B2), or 2C TCR (clone 1B2) were purchased from BD Biosciences. mAbs specific for mouse H2K^d^ (clone SF1-1.1.1), CD25 (clone pc61.5), CXCR3-biotin (clone CXCR3-173), or CD62L (clone MEL-14) were purchased from eBioscience. mAb specific for mouse CD44 (clone IM7) was from BioLegend. Biotinylated antibodies were detected using streptavidin conjugates with APC-Cy7 or PE-Cy7 purchased from BD Biosciences. mAbs specific for mouse IFN-γ (clone XMG1.2), TNF-α (clone MP6-XT22), GM-CSF (clone MP1-22E9), or IL-17A (clone TC11-18H10.1) were from BioLegend. mAbs specific for Ki67 (clone B56), Foxp3 (clone FJK-16S), Helios (clone 22F6), or T-bet (clone 4B10) were from BD Biosciences. Antibody against CHOP (clone B-3) was from Santa Cruz Biotechnology. Antibodies against phosphorylated PERK (p-PERK; 3179S) and XBP1s (12782S) were from Cell Signaling Technology. Antibody against Nrf2 (ab92946) was purchased from Abcam. Secondary antibodies PE-IgG1 (clone A85-1) and PE-IgG1 isotype control (clone MOPC-21) were purchased from BD Biosciences and BioLegend, respectively. Detailed protocols for surface staining and intracellular staining have been described previously (44, 45). Flow cytometry analysis was performed with Cytek Aurora (Cytek Biosciences), and the FACS data were analyzed using FlowJo software v10 (Tree Star).

### Histopathology.

GVHD target organs including skin, liver, lung, small intestine, and large intestine were harvested 21 days after BMT. The tissues were fixed in 10% formalin embedded in paraffin blocks, sectioned, and stained with hematoxylin and eosin. Pathology scores of tissues were blindly evaluated as previously described (44–46).

### Bioluminescent imaging.

Recipient mice receiving luciferase-expressing P815 or Raji cells were intraperitoneally injected with 200 μL luciferin purchased from Goldbio, and then anesthetized using isoflurane for evaluation of tumor growth in vivo using IVIS Spectrum CT (PerkinElmer). Data were analyzed using software purchased from PerkinElmer.

### Quantitative real-time PCR.

Total RNA of T cells purified from syngeneic and allogeneic BMT mice was extracted using Trizol reagent purchased from Thermo Fisher Scientific. RNAs were reversely transcribed into cDNA using a reverse transcription kit purchased from Bio-Rad. Quantitative real-time PCR was performed using SYBR Green (Bio-Rad). The mouse housekeeping gene, actin, was used as internal reference for data normalization. Fold changes in data shown in Figure 1B and Figure 6B were calculated using the ΔΔCt method. All primers are listed as follows (5′→3′): mouse Eif2ak3 forward (F): ATGCCGGGGCTAAGTTGTAGA; mouse Eif2ak3 reverse (R): AACGGATACGTCGTCTGGATA; mouse Syvn1-F: CCAACATCTCCTGGCTCTTCCA; mouse Syvn1-R: CAGGATGCTGTGATAAGCGTGG; mouse Sel1l-F: GGAAGTGACATCGTACCTCAGAG; mouse Sel1l-R: CTTGAACGCCTCTTCCGTAGAG; mouse Erlec1-F: GCAGGAAGAGATACTGAGAGTGC; mouse Erlec1-R: GGTTGTTCCAACAGTGAGCACTG; mouse Atf4-F: GCCTGACTCTGCTGCTTA; mouse Atf4-R: GCCTTACGGACCTCTTC; mouse Os9-F: CATCGGCTGAAACGCTACCACA; mouse Os9-R: CGGGTTCATCTACTCGGTCAATG; mouse Ddit3-F: GGAGCTGGAAGCCTGGTATG; mouse Ddit3-R: GGATGTGCGTGTGACCTCTG; mouse Man1b1-F: AGTGGACTTCAGACAGCACGGT; mouse Man1b1-R: GGATGTGCTTTGTCACTTCCTCC; mouse Edem1-F: GCTGCGTATCAGAGCATCCAGA; mouse Edem1-R: CAGCGAGTCAATCCAGGTGTTC; mouse Edem2-F: GACCCTGTGTTTGAAGATGTGGC; mouse Edem2-R: CACTTGCCAGTGAGCACATCGA; mouse Edem3-F: CCACCACAAACCGAAGCATCTC; mouse Edem3-R: GGTTCACGGATGCTCTGAGCAT.

### RNA-Seq.

Total RNA of T cells purified from WT and PERK-cKO mice and stimulated with allogeneic APCs or anti-CD3/CD28 was extracted using Trizol reagent (Thermo Fisher Scientific). The RNA concentration was evaluated by NanoDrop Microvolume Spectrophotometers (Thermo Fisher Scientific). RNA integrity was determined by running of electrophoresis. Data were analyzed by DESeq2, pheatmap, and clusterProfiler packages using R and RStudio software.

### Immunoprecipitation and Western blot.

Immunoprecipitation was performed by addition of specific antibodies into cell lysates for rotation overnight in 4°C. Next, protein A/G beads (Santa Cruz Biotechnology) were added for a rotation of 4–6 hours at 4°C. The samples were then boiled with loading buffer for 10 minutes at 98°C after washing 5 times with Thermo Fisher Scientific Pierce IP lysis buffer.

Cells were harvested and lysed using RIPA lysis buffer or IP lysis buffer supplemented with protease inhibitor cocktail (MilliporeSigma). Protein concentrations were measured by BCA Protein Assay Kit (Thermo Fisher Scientific), and equivalent proteins were subjected to SDS-PAGE gel and then transferred to PVDF membrane. After blocking with 5% nonfat milk, the membrane was incubated with specific primary antibodies overnight at 4°C. Then, secondary antibodies were incubated for 1 hour at room temperature after washing 3 times. SuperSignal West Pico PLUS Chemiluminescent Substrate (Thermo Fisher Scientific) was used to visualize the immunoreactivity bands. Anti–mouse PERK (31292S), anti–mouse p-PERK (3179S), anti–human PERK (5683S), anti–mouse XBP1s (12782S), anti–mouse/human GAPDH (2118S), and anti–mouse/human β-actin (8457T) antibodies were purchased from Cell Signaling Technology. Anti–mouse GADD153 (CHOP, sc-7351) was purchased from Santa Cruz Biotechnology. Anti–mouse/human SEL1L (ab78298) and anti–mouse/human ERLEC1 (ab181166) antibodies were purchased from Abcam.

### Statistics.

GraphPad Prism 9.0 was used to generate figures and calculate statistics. Survival comparison was analyzed by log-rank (Mantel-Cox) test. Both GVHD scores and body weight loss comparisons were analyzed by non-parametric Mann-Whitney *U* test. Multiple comparisons were determined by 1-way or 2-way ANOVA test. Comparisons between 2 groups were analyzed by 2-tailed unpaired Student’s *t* test. Data are shown as mean ± SD. *P* less than 0.05 was considered statistically significant (*P < 0.05, **P < 0.01, ***P < 0.001, ****P < 0.0001).

### Study approval.

Experimental protocols were approved by the IACUC of Medical College of Wisconsin (AUA00007677, AUA00007641).

### Data availability.

The values corresponding to all data points shown in graphs and values behind any reported means are available in the Supporting Data Values Excel file. All data generated or analyzed during this study are included in Figures 1–7 and Supplemental Figures 1–16. Raw data of RNA-Seq were deposited in the NCBI’s Gene Expression Omnibus database (GEO GSE307389).

## Author contributions

QC, HJC, and XZY conceived and designed this project. QC and HJC performed the experiments and analyzed data. QC drafted the manuscript. YW contributed to discussing BMT experiments and revising the manuscript. XY, AP, LT, MH, and DF contributed to the in vivo experiments. RA contributed to the human T cell in vitro experiments. CL evaluated pathology scores of tissues blindly. XZY interpreted the data, revised the manuscript, and supervised the work.

## Funding support

This work is the result of NIH funding, in whole or in part, and is subject to the NIH Public Access Policy. Through acceptance of this federal funding, the NIH has been given a right to make the work publicly available in PubMed Central.

- NIH grants R01CA258440, R01HL163584, and R21CA263140 (to XZY).
- Institutional start-up funds from the Cancer Center and Department of Microbiology & Immunology, Medical College of Wisconsin (to XZY).

## Supplementary Material

Supplemental data

Unedited blot and gel images

Supporting data values

## Version 1 09/30/2025

In-Press Preview

## Version 2 12/01/2025

Electronic publication
