# Supplementary material for: ER stress sensor PERK promotes T cell pathogenicity in GVHD by regulating ER-associated degradation
Source: J Clin Invest. 2025 Sep 30;135(23):e190958. doi: 10.1172/JCI190958 (PMC12646671; doi:10.1172/JCI190958)
Supplement: Supplemental data [file jci-135-190958-s246.pdf]

Supplemental Figure 1

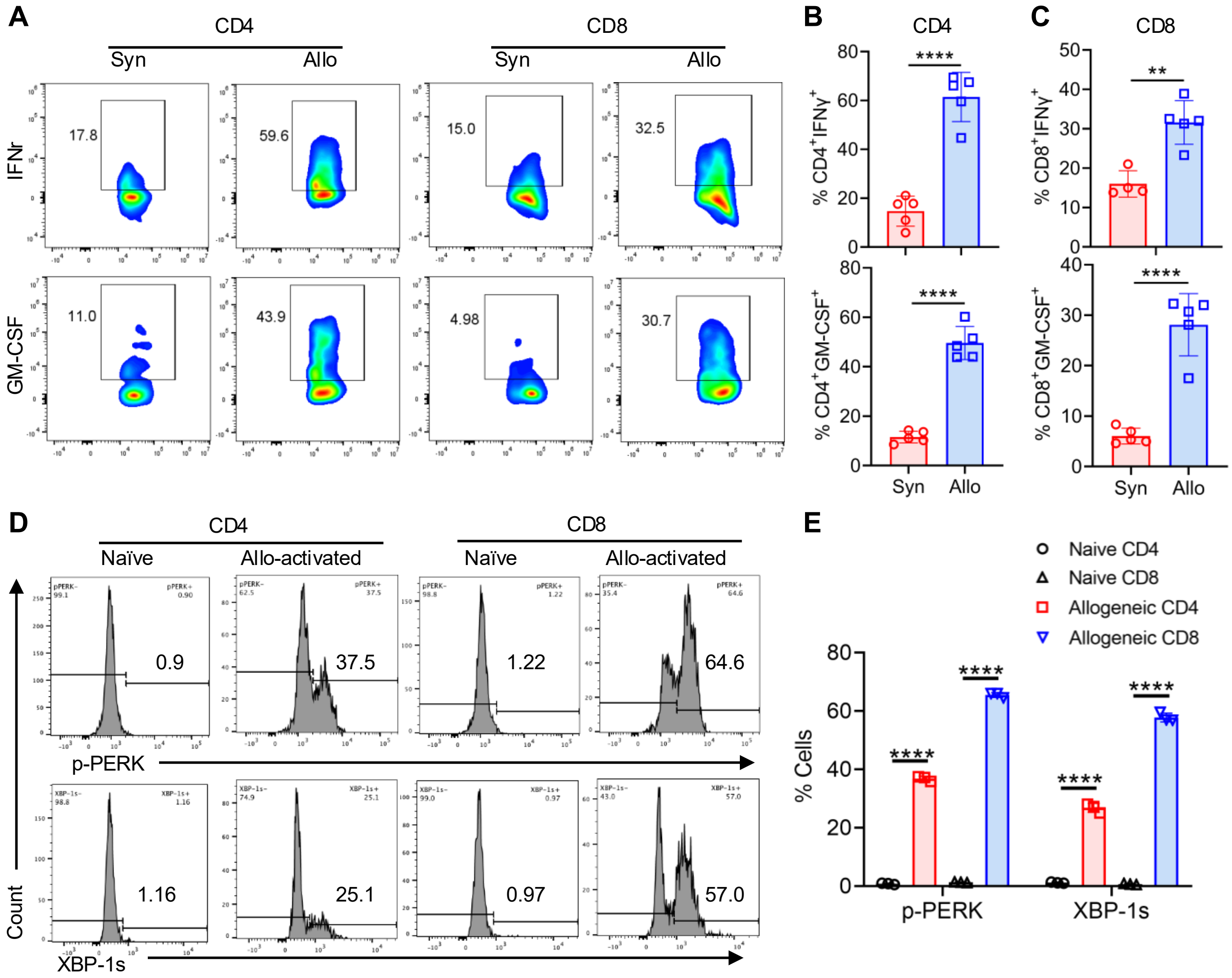

Supplemental Figure 1. Allogeneic stimulation activates ER stress signaling in T cells. (**A-C**) Lethally irradiated WT B6 (Ly5.1, syngeneic) and BALB/c (allogeneic) recipients were transferred with T cells (1.25 X 10^6) isolated from normal B6 (Ly5.2) donors plus BM cells (4 X 10^6) from Rag1-KO donors, n = 4-5 per group. The expression levels of IFNγ and GM-CSF in donor $CD4^+$ and $CD8^+$ T cells were analyzed by flow cytometry (**A**). Percentages of $IFN\gamma^+CD4^+$, $GM\text{-}CSF^+CD4^+$ among gated $H2K^{b+}CD4^+$ T cells are shown (**B**). Percentages of $IFN\gamma^+CD8^+$, $GM\text{-}CSF^+CD8^+$ among gated $H2K^{b+}CD8^+$ T cells are shown (**C**). Total T cells isolated from WT B6 mice were stimulated with allogeneic APCs from BALB/c mice for 4 days. Naïve or activated T cells were stained for expression of phosphorylated PERK (p-PERK) and XBP-1s and detected using flow cytometry (**D**). Percentages of $p\text{-}PERK^+CD4^+$, $p\text{-}PERK^+CD8^+$, $XBP\text{-}1s^+CD4^+$, and $XBP\text{-}1s^+CD8^+$ T cells are displayed (**E**). Data in panels **B**, **C**, **E** are presented as mean ± SD, significance was determined using a two-tailed unpaired Student's *t* test. *P < .05, **P < .01, ***P < .001, ****P < .0001.

Supplemental Figure 2

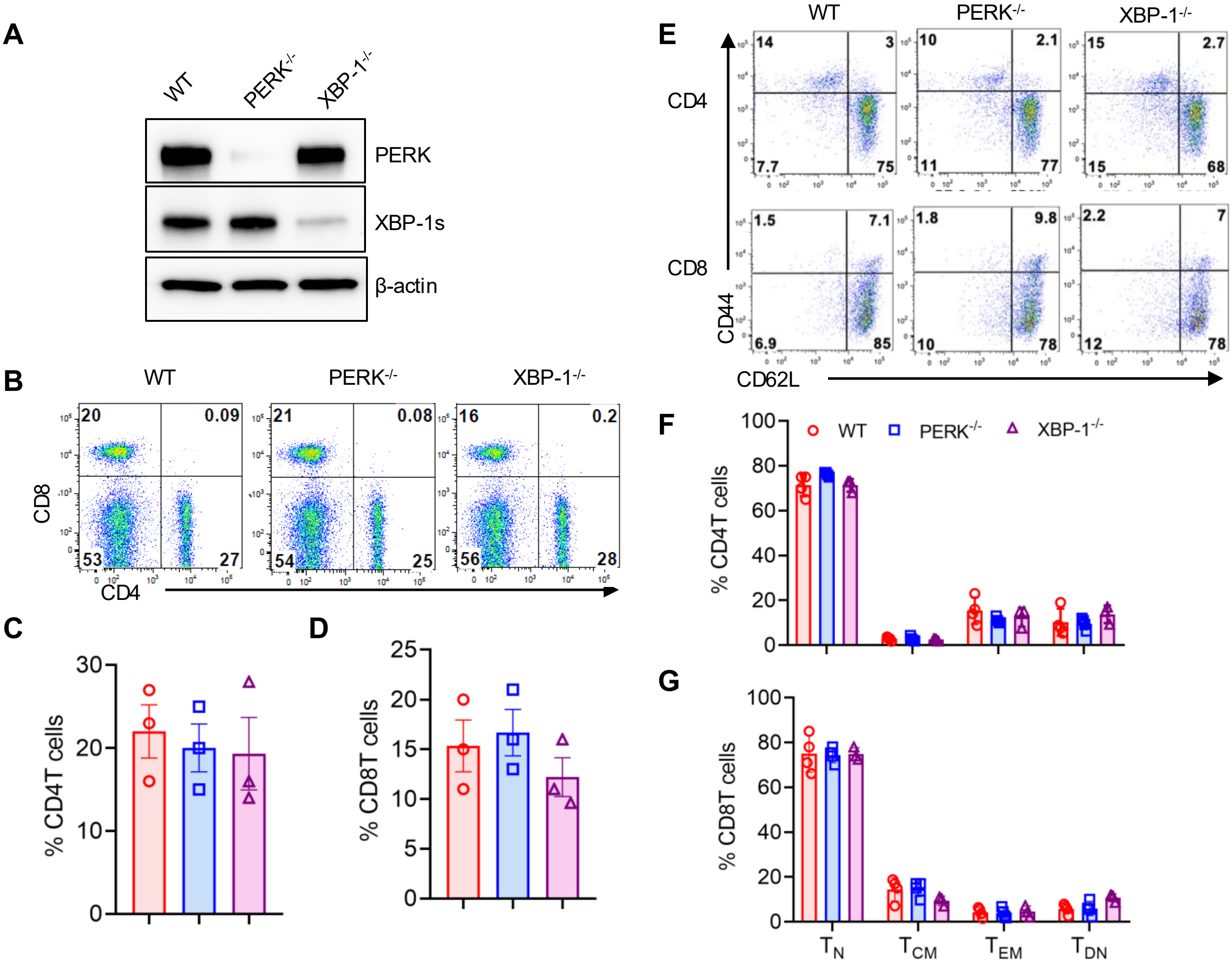

Supplemental Figure 2. PERK and XBP1 do not affect T-cell development. T cells isolated from WT B6, PERK cKO, XBP1 cKO mice were stimulated with allogeneic APCs from BALB/c mice for 4 days, then the protein levels PERK, XBP-1s and β-actin in T cells were evaluated by western blot (**A**). (**B-G**) Spleens were harvested from B6 WT, PERK cKO or XBP1 cKO mice, n = 3 per group. $CD4^+$, $CD8^+$ T cells were analyzed by flow cytometry (**B**). Percentages of $CD4^+$ (**C**) or $CD8^+$ T cells (**D**) among gated lymphocytes are displayed. $CD44^-CD62L^+$ (naïve T, T_N), $CD44^+CD62L^+$ (central memory T, T_{CM}), $CD44^+CD62L^-$ (effector memory T, T_{EM}), $CD44^-CD62L^-$ (double negative T, T_{DN}) were analyzed by flow cytometry (**E**). Percentages of T_N, T_{CM}, T_{EM}, T_{DN} among gated $CD4^+$ T cells are displayed (**F**). Percentages of T_N, T_{CM}, T_{EM}, T_{DN} among gated $CD8^+$ T cells are displayed (**G**). Data in panels **C**, **D**, **F**, **G** are shown as mean ± SD, significance was conducted using a one-way ANOVA test.

Supplemental Figure 3

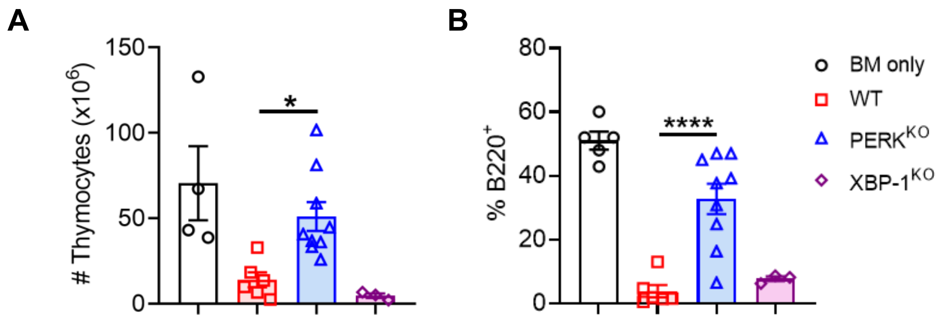

Supplemental Figure 3. Deficiency of PERK in donor T cells increases thymocyte recovery and B-cell reconstitution. (**A-B**) Lethally irradiated BALB/c mice were transferred with TCD-BM cells (5 X 10^6) alone or together with T cells (1.25 X 10^6) from WT B6 or PERK cKO or XBP1 cKO donors. (**A**) Absolute numbers of thymocytes of the recipients are shown on day 14 after BMT. (**B**) Percentages of $B220^+$ cells among gated $H2K^{b+}$ lymphocytes in the recipient spleens are shown on day 14 after BMT. Data in panels **A-B** are represented as mean ± SD, significance was determined using a one-way ANOVA test. **P* < .05, ***P* < .01, ****P* < .001.

Supplemental Figure 4

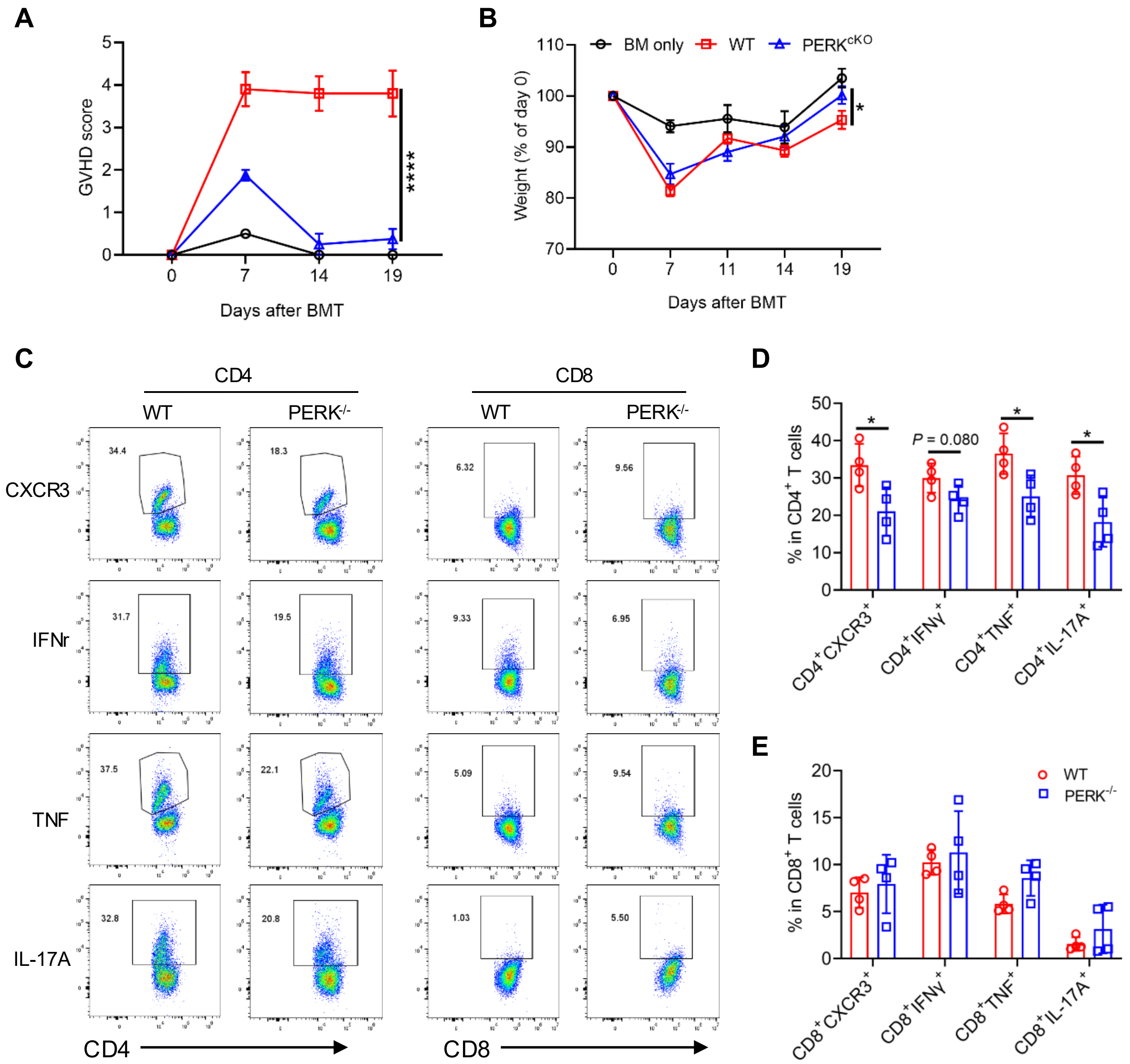

Supplemental Figure 4. PERK deficiency in donor T cells inhibits GVHD and suppresses CD4⁺ T cells allogeneic responses. (**A-E**) Lethally irradiated BALB/c mice (900 cGy) were injected with TCD-BM cells (5 X 10^6) alone or together with T cells (1.25 X 10^6) isolated from WT B6 or PERK cKO donors, n = 2 fro BM only, n = 4-5 for WT or PERK cKO. GVHD scores (**A**), body weight (**B**) of BALB/c recipients were monitored through 21 days post-BMT. (**C**) $CXCR3^+CD4^+$, $IFN\gamma^+CD4^+$, $TNF\alpha^+CD4^+$, IL-17A$^+$CD4$^+$ T cells and $CXCR3^+CD8^+$, $IFN\gamma^+CD8^+$, $TNF\alpha^+CD8^+$, IL-17A$^+$CD8$^+$ T cells in recipient intestines were analyzed by flow cytometry. (**D**) Percentages of $CXCR3^+CD4^+$, $IFN\gamma^+CD4^+$, TNF^+CD4^+, and IL-17$^+$CD4$^+$ T cells among gated $H2K^{b+}CD4^+$ T cells are displayed. (**E**) Percentages of $CXCR3^+CD8^+$, $IFN\gamma^+CD8^+$, TNF^+CD8^+, and IL-17$^+$CD8$^+$ T cells among gated $H2K^{b+}CD8^+$ T cells are displayed. Nonparametric Mann–Whitney U tests were conducted to compare groups in panels **A**, **B**. Data in panels **D**, **E** are represented as mean ± SD with biological replicates, significance was determined using a two-tailed unpaired Student's *t* test, *$P < .05$, **$P < .01$, ***$P < .001$, ****$P < .0001$.

Supplemental Figure 5

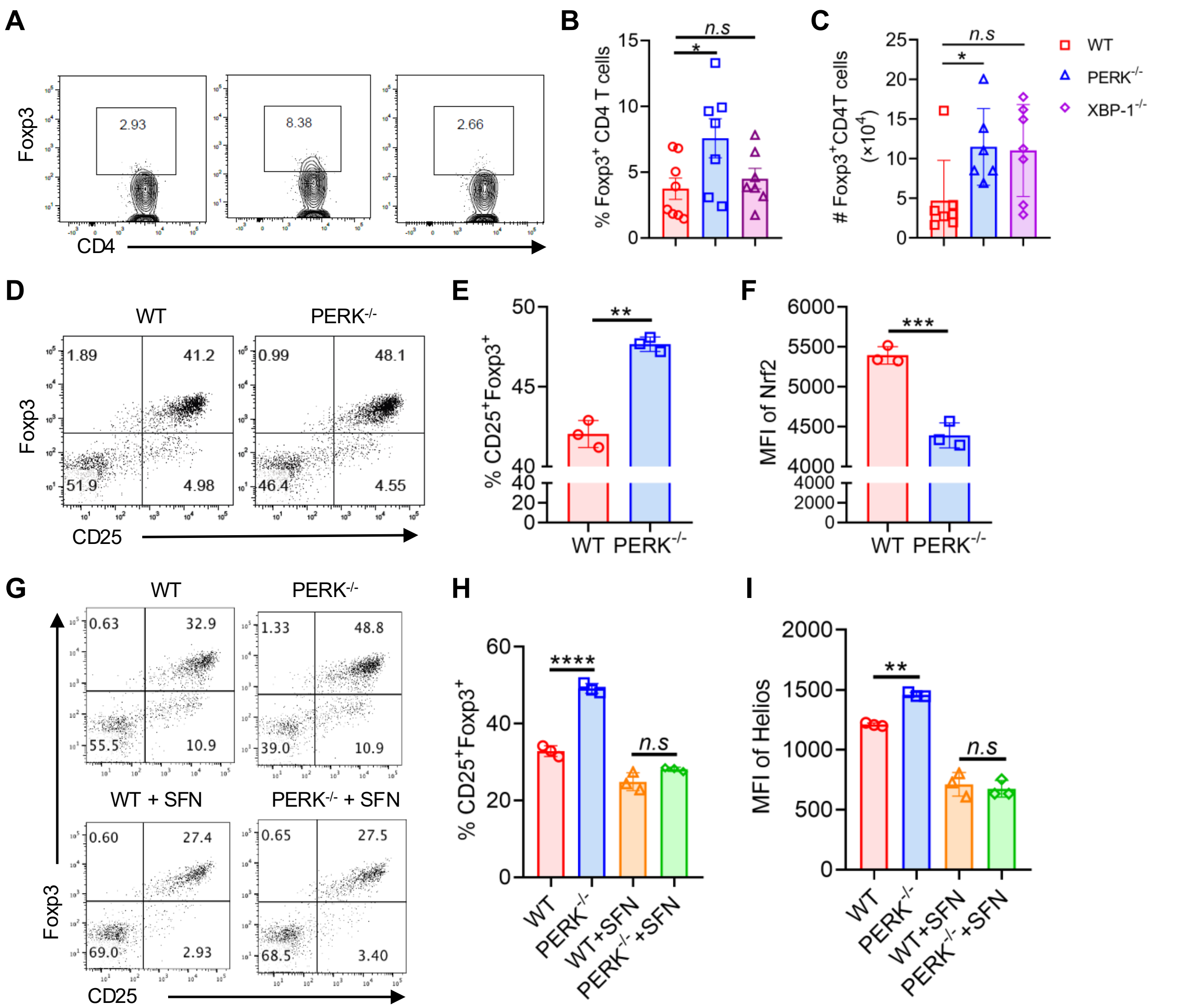

Supplemental Figure 5. PERK inhibits Treg generation through regulating Nrf2 signaling pathway. (**A-C**) Lethally irradiated BALB/c mice were transferred with TCD-BM cells (5 X 10^6) alone or along with T cells (1.25 X 10^6) from B6 WT, PERK cKO or XBP1 cKO donors. (**A**) Splenic $CD4^+Foxp3^+$ cells in the recipients were analyzed by flow cytometry on day 14 after BMT. Percentages (**B**) and absolute numbers (**C**) of splenic $Foxp3^+CD4^+$ T cells among gated $H2K^{b+}CD4^+$ T cells in the recipients are displayed. (D-F) $CD25^-CD4^+$ T cells isolated from WT B6 or PERK cKO mice were stimulated with anti-CD3 (1 µg/ml) and polarized into iTreg with IL-2 (2 ng/ml) and TGFβ (5 ng/ml). (**D**) $CD25^+Foxp3^+$ cells in $CD4^+$ T cells were analyzed by flow cytometry. (**E**) Percentages of $CD25^+Foxp3^+$ cells among gated $CD4^+$ T cells are displayed. (**F**) Mean Fluorescent Intensity (MFI) of Nrf2 among gated $CD4^+$ T cells is displayed. (**G-I**) $CD25^-CD4^+$ T cells isolated from WT B6 or PERK cKO mice were stimulated with anti-CD3 under iTreg-polarization condition and treated with or without sulforaphane (SFN). (**G**) $CD25^+Foxp3^+$ cells in $CD4^+$ T cells were analyzed by flow cytometry. (**H**) Percentages of $CD25^+Foxp3^+$ cells among gated $CD4^+$ T cells are displayed. (**I**) MFI of Helios among gated $CD4^+$ T cells is displayed. Data in panels **B**, **C, E, F, H, I** are represented as mean ± SD, significance in panels **B**, **C** was analyzed using a one-way ANOVA test, significance in panels **E**, **F** was determined using a two-tailed unpaired Student's *t* test, a two-way ANOVA test was used to compare groups in panels **H**, **I.** $^*P < .05$, $^{**}P < .01$, $^{***}P < .001$, $^{****}P < .0001$.

Supplemental Figure 6

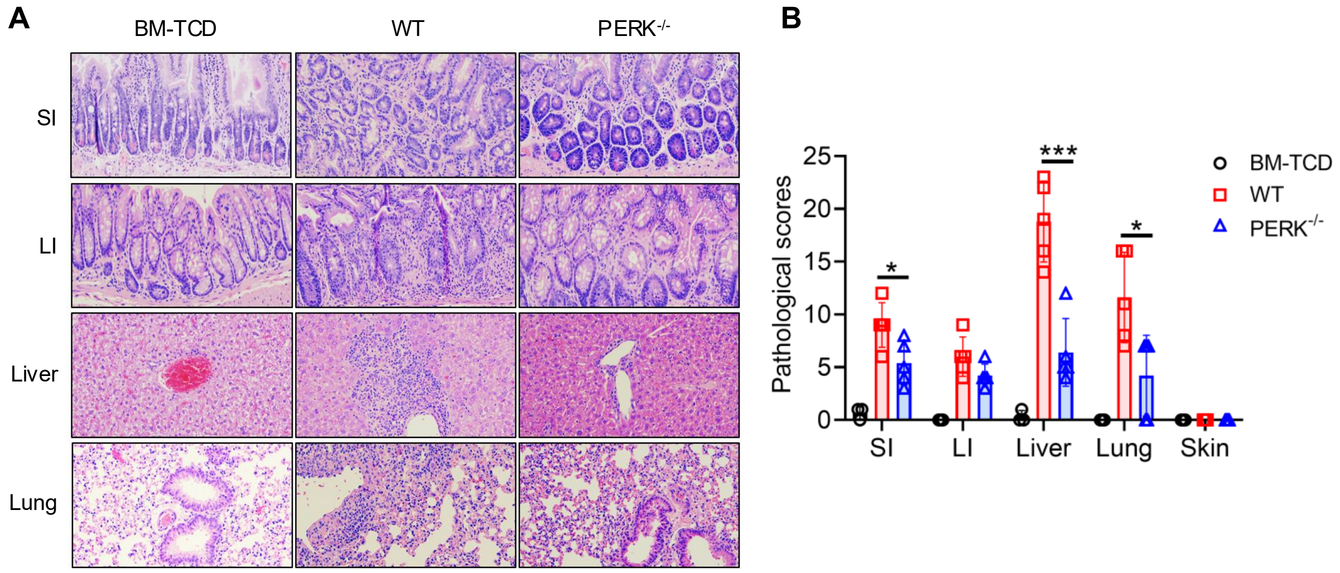

Supplemental Figure 6. PERK-deficiency in donor T cells suppress GVHD. (**A-B**) Lethally irradiated BDF1 mice were injected with TCD-BM cells (5 X 10^6) alone or together with CD25-removed T cells (3 X 10^6) from WT or PERK cKO donors, n = 3 for BM-TCD, n = 5 for WT or PERK cKO. (**A**) Pathology of small intestine (SI) and large intestine (LI), liver and lung of BALB/c recipients was analyzed on the tissues with HE staining. (**B**) Pathology scores of small and large intestines, liver, lung, and skin are displayed. Data in panel B are represented as mean ± SD, significance was analyzed using a one-way ANOVA test. **P* < .05, ***P* < .01, ****P* < .001.

Supplemental Figure 7

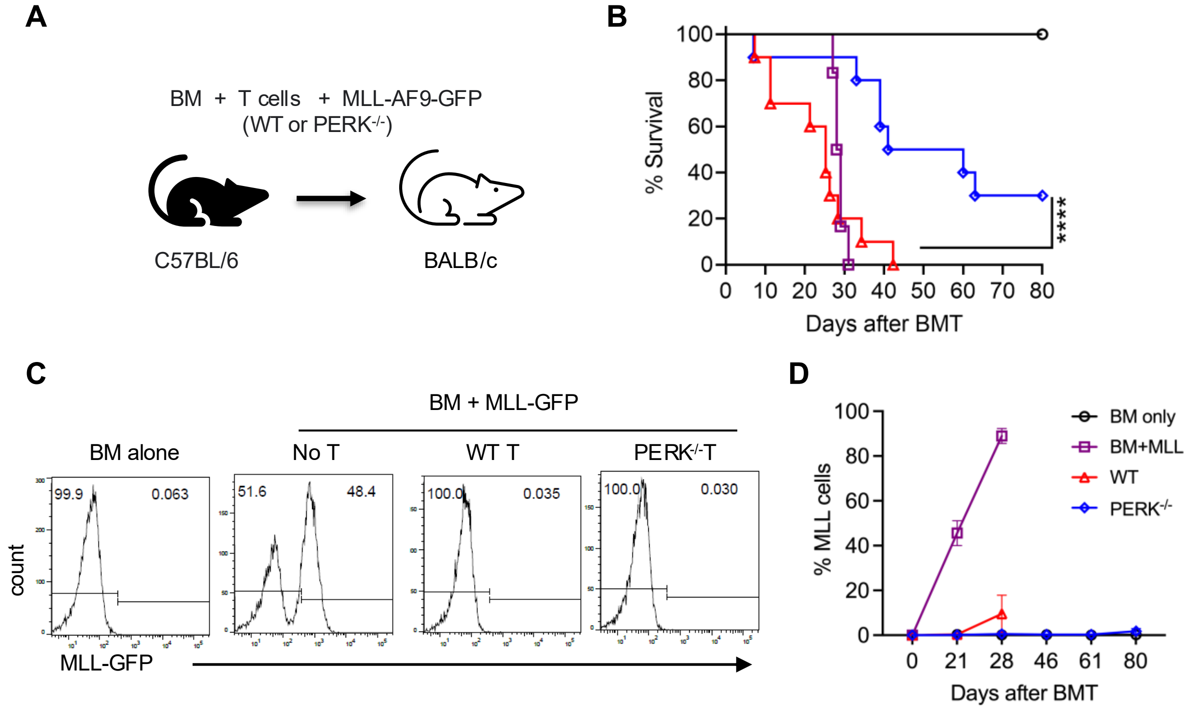

Supplemental Figure 7. PERK-deficient donor T cells induces milder GVHD while preserving the GVL effect. (**A-D**) Lethally irradiated BALB/c mice were injected with TCD-BM cells (5 X 10^6) alone or along with MLL-GFP cells with or without T cells (1.25 X 10^6) purified from WT B6 or PERK cKO donors, n = 3 for BM only, n = 6 for BM with MLL, n = 10 for WT or PERK cKO (**A**) Schematic model of BMT. (**B**) Survival of recipient mice was monitored through 80 days post-BMT. (**C**) MLL-GFP$^+$ cells in peripheral blood of the recipient mice after BMT were evaluated by flow cytometry. (**D**) Percentages of MLL-GFP$^+$ cells in recipient peripheral blood after BMT are displayed. The survival curve in panel **B** was analyzed by Log-rank (Mantel-Cox) test. **P* < .05, ***P* < .01, ****P* < .001, *****P* < .0001.

Supplemental Figure 8

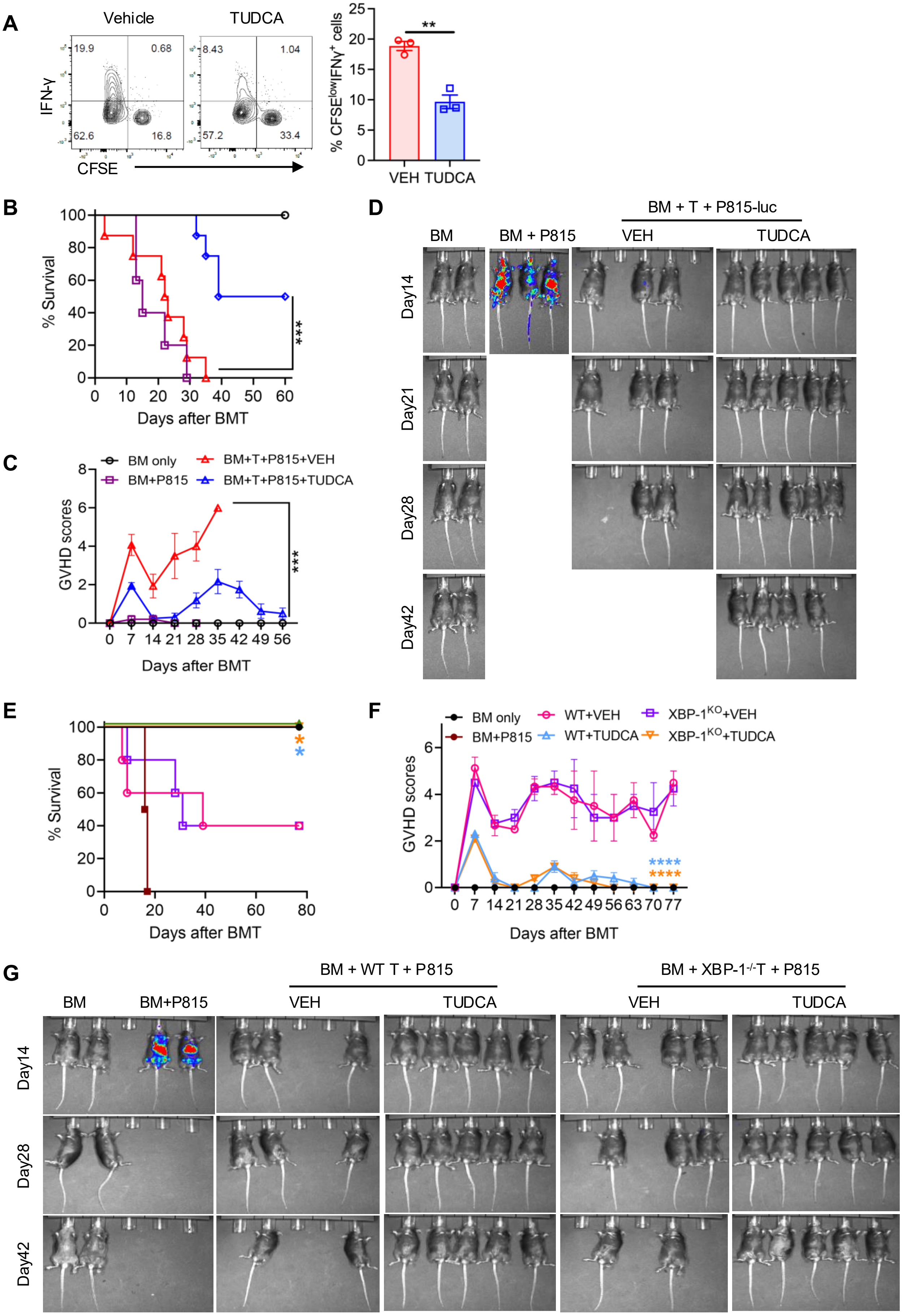

Supplemental Figure 8. Targeting ER stress reduces T-cell mediated GVHD while preserving the GVL effect. (**A**) T cells isolated from WT B6 mice were labeled with CFSE and stimulated with allogeneic APCs from BALB/c mice for 4 days. The levels of IFNγ in $CD4^+$ T cells were analyzed by flow cytometry. Percentages of $CFSE^{low}$ $IFN\gamma^+$ among gated $H2K^{b+}CD4^+$ T cells are shown. (**B-D**) Lethally irradiated BDF1 mice were transferred with TCD-BM cells (5 X 10^6) alone or together with P815-luc cells (5000) with or without CD25-removed T cells (3 X 10^6) from normal B6 mice. Vehicle or TUDCA (10 mg/kg) was i.p injected into BDF1 recipients every other day for 2 weeks post-BMT, n = 4 for BM only, n = 5 for BM with P815, n = 8 for VEH or TUDCA. Survival (**B**) and clinical scores (**C**) were monitored through 80 days post-BMT. Tumor growth in BDF1 recipients was monitored using BLI (**D**). (**E-G**) BMT was set up as described above, Vehicle or TUDCA (10 mg/kg) was i.p injected into BDF1 recipients every other day for 2 weeks post-BMT, n = 2 for BM only or P815, n = 5 for WT or XBP1 cKO with VEH or TUDCA. Survival (**E**) and clinical scores (**F**) were monitored through 80 days post-BMT. Tumor growth in BDF1 recipients was monitored using BLI (**G**). Log-rank (Mantel-Cox) test (**B**, **E**) and non-parametric Mann-Whitney U test (**C**, **F**) were used to compare groups. Data in panel **A** are represented as mean ± SD, significance was determined using a two-tailed unpaired Student's *t* test, $^*P < .05$, $^{**}P < .01$, $^{***}P < .001$, $^{****}P < .0001$.

Supplemental Figure 9

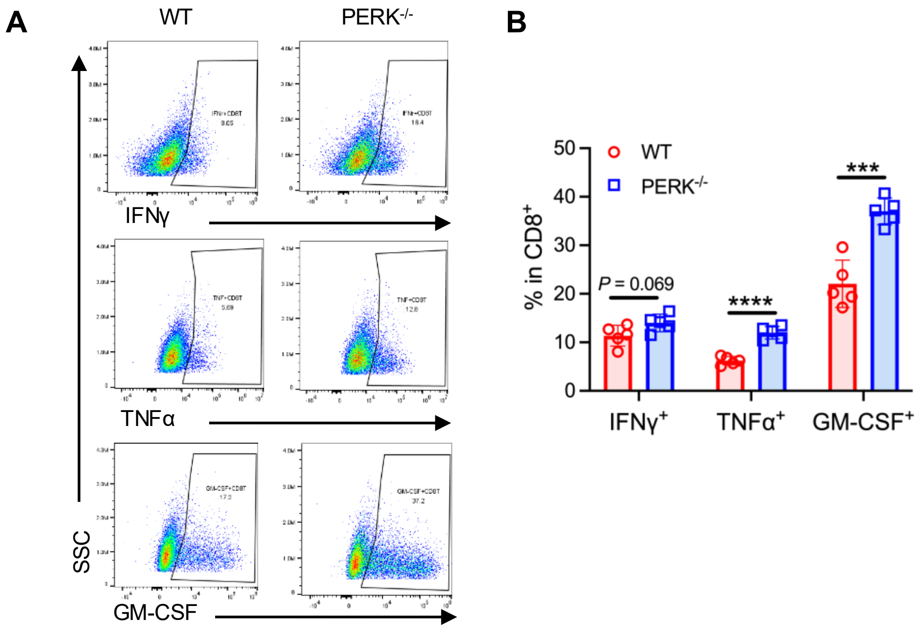

Supplemental Figure 9. PERK inhibits CD8 T-cell allogenic responses *in vivo.* (**A-B**) Lethally irradiated BALB/c mice were injected with TCD-BM cells (5 X 10^6) along with $CD8^+$ T cells (2.5 X 10^6) isolated from WT B6 or PERK cKO donors, n = 5 per group. (**A**) $IFN\gamma^+CD8^+$, $TNF\alpha^+CD8^+$, $GM\text{-}CSF^+CD8^+$ T cells in livers of recipient mice were analyzed on day 14 after BMT by flow cytometry. (**B**) Percentages of $IFN\gamma^+CD8^+$, $TNF\alpha^+CD8^+$, $GM\text{-}CSF^+CD8^+$ T cells among gated $H2K^{b+}CD8^+$ T cells are displayed. Data in panel B are represented as mean $\pm$ SD with biological replicates, significance was determined using a two-tailed unpaired Student's *t* test, *$P < .05$, **$P < .01$, ***$P < .001$, ****$P < .0001$.

Supplemental Figure 10

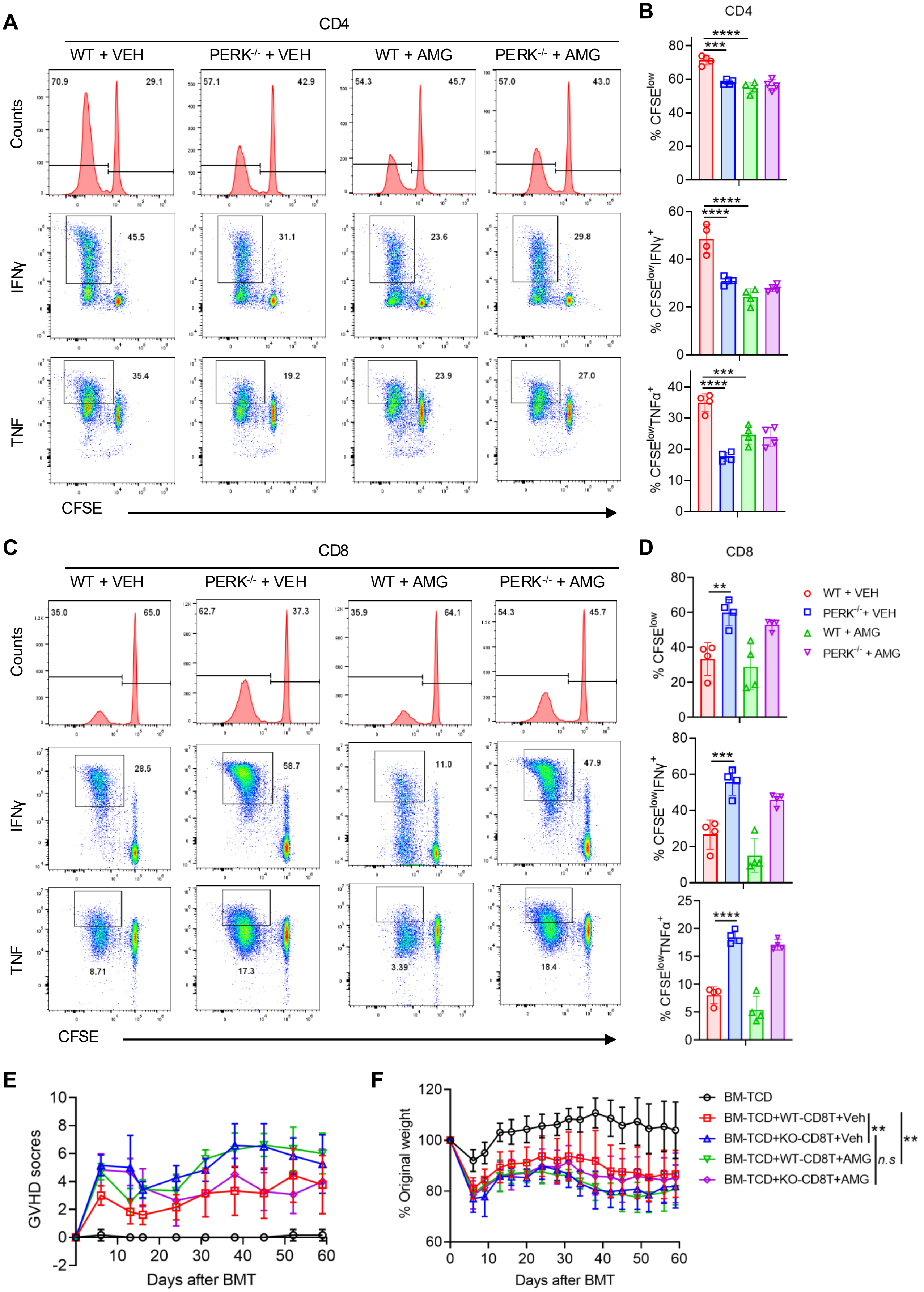

Supplemental Figure 10. PERK differentially regulates CD4 and CD8 T-cell responses to alloantigens. (**A-B**) $CD4^+$ T cells isolated from WT or PERK cKO mice were stimulated with allogeneic APCs from BDF1 mice and treated with Vehicle or AMG44 (2 μM) for 4 days, proliferation ($CFSE^{low}$) of $CD4^+$ T cells, and levels of pro-inflammatory cytokines (IFNγ, TNFα) in $CD4^+$ T cells were analyzed using flow cytometry (**A**). Percentages of $CFSE^{low}CD4^+$, $CFSE^{low}IFN\gamma^+CD4^+$, $CFSE^{low}TNF\alpha^+CD4^+$ T cells among gated $H2K^{d-}CD4^+$ T cells are shown (**B**). (**C-D**) $CD8^+$ T cells isolated from WT or PERK cKO mice were stimulated with allogeneic APCs from BDF1 mice and treated with Vehicle or AMG44 (2 μM) for 4 days, proliferation ($CFSE^{low}$) of $CD8^+$ T cells, and levels of pro-inflammatory cytokines (IFNγ, TNFα) in $CD8^+$ T cells were analyzed by flow cytometry (**C**). Percentages of $CFSE^{low}CD8^+$, $CFSE^{low}IFN\gamma^+CD8^+$, $CFSE^{low}TNF\alpha^+CD8^+$ T cells among gated $H2K^{d-}$ $CD8^+$ T cells are shown (**D**). (**E-F**) Lethally irradiated BALB/c recipients (900 cGy) were injected with TCD-BM cells (5 X 10^6) alone or along with $CD8^+$ T cells (2.5 X 10^6) from WT B6 or PERK cKO donors and CD25-removed $CD4^+$ T cells (0.5 X 10^6) from WT B6 mice. Recipient mice were injected with vehicle or AMG44 (5 mg/kg) every other day for 2 weeks after BMT. GVHD scores (**E**), body weight (**F**) of BALB/c recipients were monitored through 60 days post-BMT, n = 6 for BM-TCD, n = 10 for WT or PERK cKO with VEH or AMG combined from 2 replicate experiments. Nonparametric Mann–Whitney U tests (**E**, **F**) were conducted to compare groups. Data in panels **B**, **D** are represented as mean ± SD with biological replicates, significance was analyzed using a two-way ANOVA test. *$P < .05$, **$P < .01$, ***$P < .001$, ****$P < .0001$.

Supplemental Figure 11

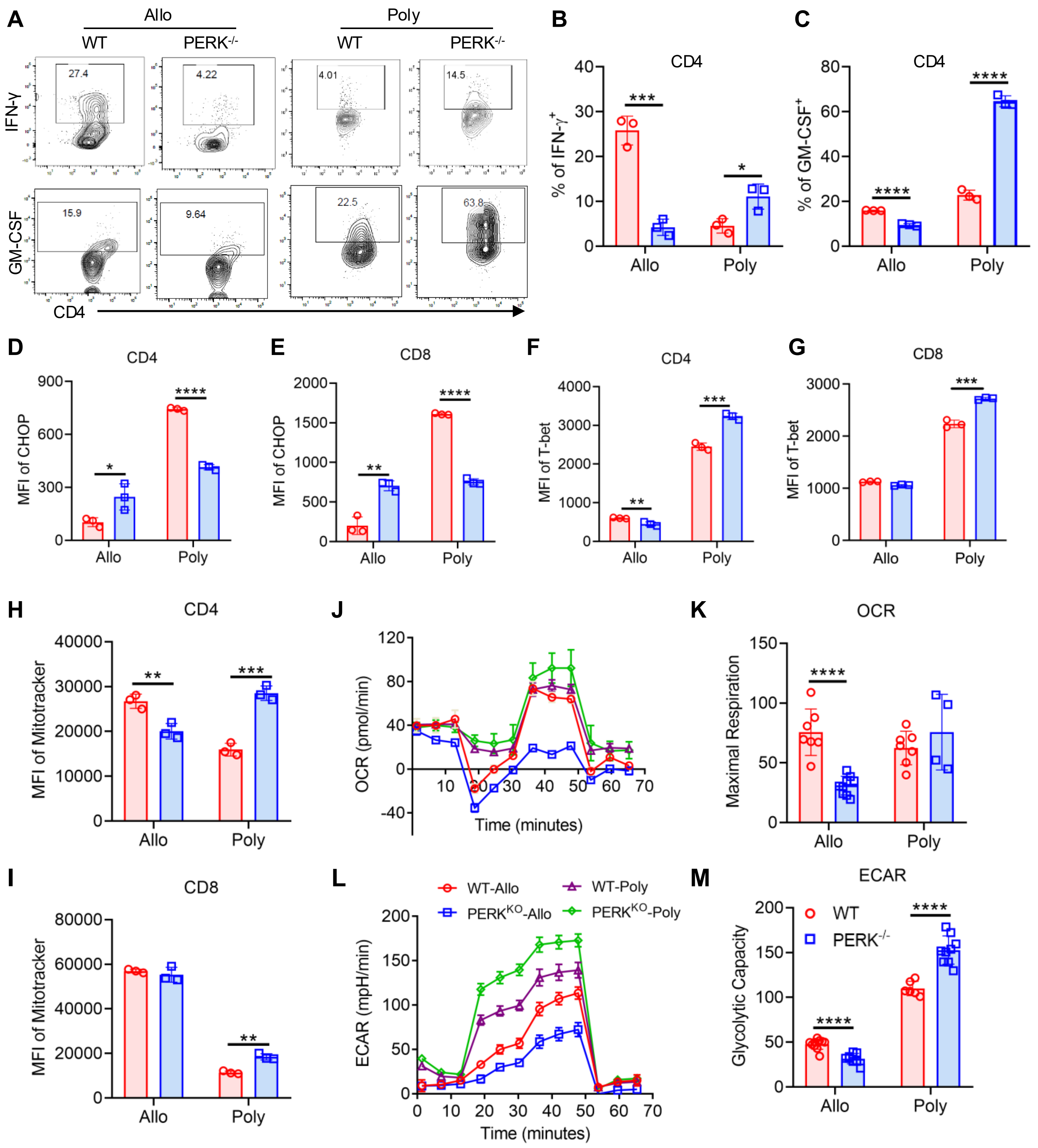

Supplemental Figure 11. PERK distinctly regulates T cell allogenic and polyclonal responses. (**A-C**) Total T cells isolated from B6 WT or PERK cKO mice were stimulated with allogenic APCs from BALB/c mice for 4 days or anti-CD3/CD28 (2 μg/ml) for 3 days. (**A**) $IFN\gamma^{+}CD4^{+}$, GM-$CSF^{+}CD4^{+}$ T cells were analyzed by flow cytometry. Percentages of $IFN\gamma^{+}$ (**B**) or GM-CSF^{+} (**C**) among gated $CD4^{+}$ T cells are displayed. (**D-I**) T cells isolated from B6 WT or PERK cKO mice were stimulated with allogenic APCs for 4 days or anti-CD3/CD28 (2 μg/ml) for 3 days. CHOP and T-bet protein levels in $CD4^{+}$ or $CD8^{+}$ T cells were evaluated by flow cytometry. MFIs of CHOP among gated $CD4^{+}$ (**D**) or $CD8^{+}$ (**E**) T cells are displayed. MFIs of T-bet among gated $CD4^{+}$ (**F**) or $CD8^{+}$ (**G**) T cells are displayed. MFIs of Mito-tracker among gated $CD4^{+}$ (**H**) or $CD8^{+}$ (**I**) T cells were detected with flow cytometry. (**J-M**) T cells isolated from WT B6 or PERK cKO mice were stimulated with allogenic APCs from BALB/c mice for 4 days or anti-CD3/CD28 (2 μg/ml) for 3 days, and then T cells isolated were analyzed by using a Seahorse XF96e machine. OXPHOS or glycolysis and shifts in T cells were determined through evaluating OCR (**J**) and ECAR (**K**). Maximal respiration (**L**) or glycolytic capacity (M) of T cells are displayed. Data in panels **B-I**, **K**, **M** are represented as mean ± SD with biological replicates, significance was determined using a two-way ANOVA test. **P* < .05, ***P* < .01, ****P* < .001, *****P* < .0001.

Supplemental Figure 12

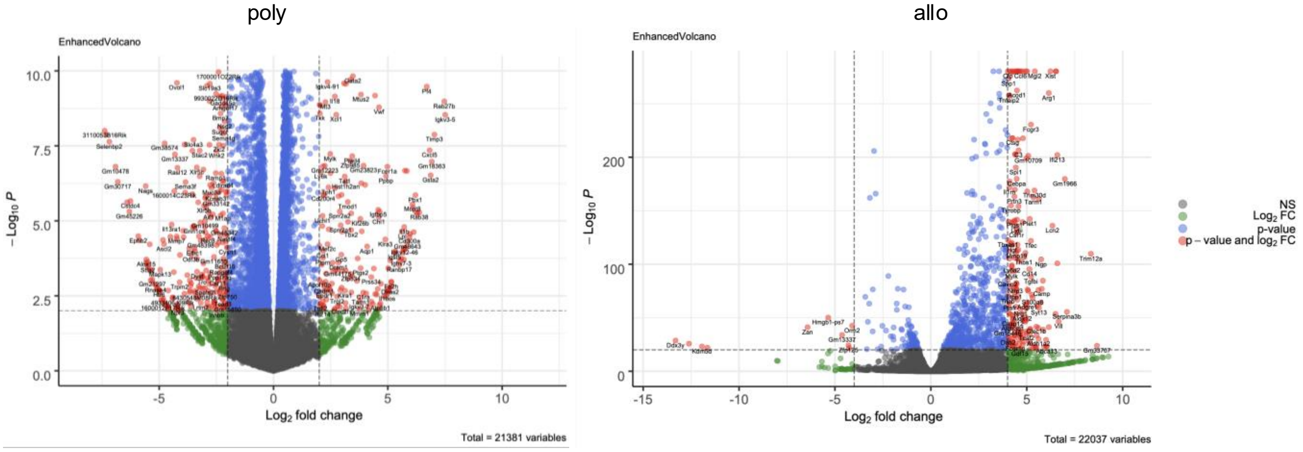

Supplemental Figure 12. Differentially expressed genes in PERK-deficient vs. WT T cells (KO vs. WT) after polyclonal or allogeneic stimulation. A volcano plot showing all the differentially expressed transcripts in PERK-deficient T cells after polyclonal or allogeneic stimulation.

Supplemental Figure 13

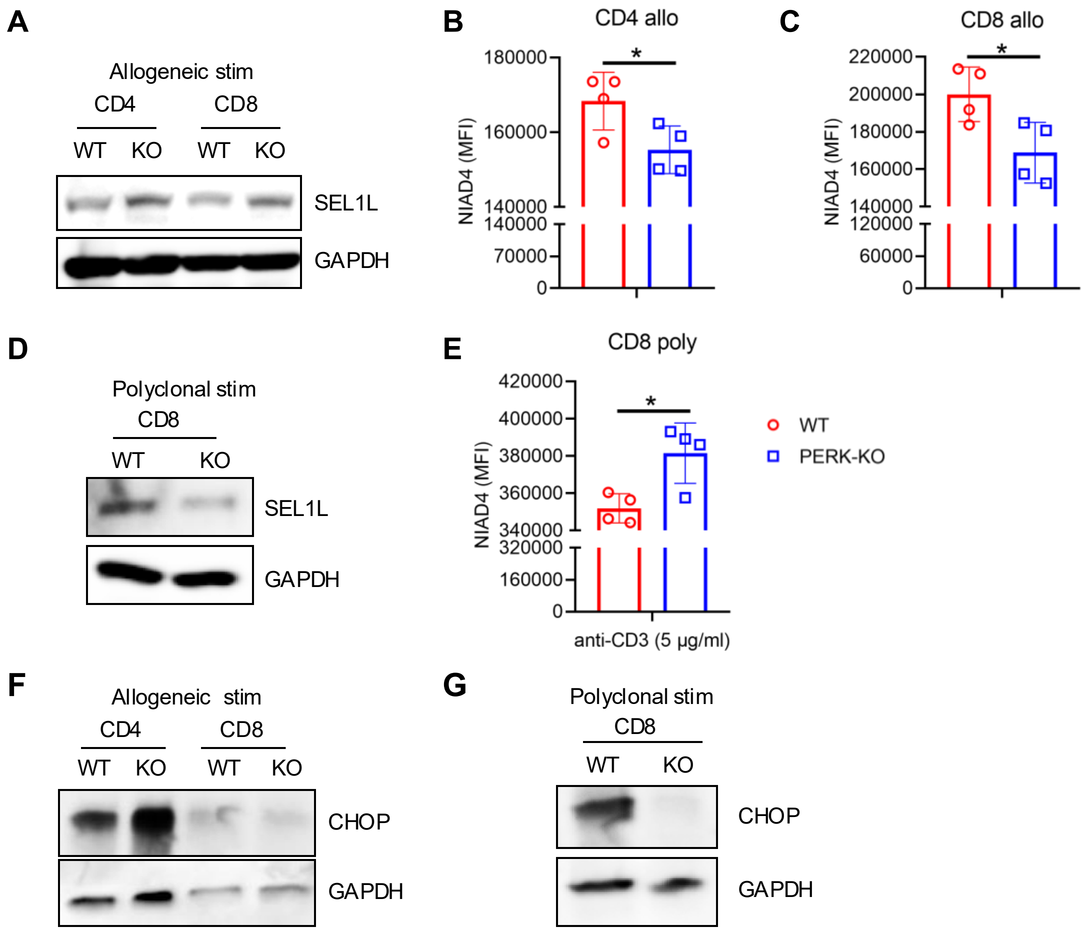

Supplemental Figure 13. PERK regulates SEL1L protein levels and the formation of aggregates. (**A-C**) $CD4^+$ or $CD8^+$ T cells isolated separately from B6 WT or PERK cKO mice were stimulated with allogeneic APCs from BDF1 mice for 4 days, then protein levels of SEL1L and GAPDH in allogeneic $CD4^+$ T or $CD8^+$ T cells were evaluated with western blot (**A**). (**B-C**) Amyloid β-protein aggregates levels were analyzed by flow cytometry after incubating with NIAD4 (10 μM). MFIs of NIAD4 among gated $H2K^d$-$CD4^+$ (**B**) or $H2K^d$-$CD8^+$ (**C**) T cells are displayed. (D-E) $CD8^+$ T cells isolated from WT or PERK cKO mice were stimulated with anti-CD3/CD28 (2 μg/ml) for 3 days. (**D**) Protein levels of SEL1L and GAPDH in $CD8^+$ T cells were detected with western blot. (**E**) MFI of NIAD4 among gated CD8 $^+$ T cells is displayed. (**F**) Protein levels of CHOP and GAPDH in $CD4^+$ or $CD8^+$ T cells isolated from WT B6 or PERK KO mice and stimulated with allogeneic APCs 4 days were evaluated by western blot. (**G**) $CD8^+$ T cells isolated from B6 WT or PERK cKO mice and stimulated with anti-CD3/CD28 (2 μg/ml) for 3 days. Protein levels of CHOP and GAPDH were detected with western blot. Data in panels **B**, **C**, **E** are represented as mean ± SD with biological replicates, significance was determined using a two-tailed unpaired Student's *t* test. *$P < .05$.

Supplemental Figure 14

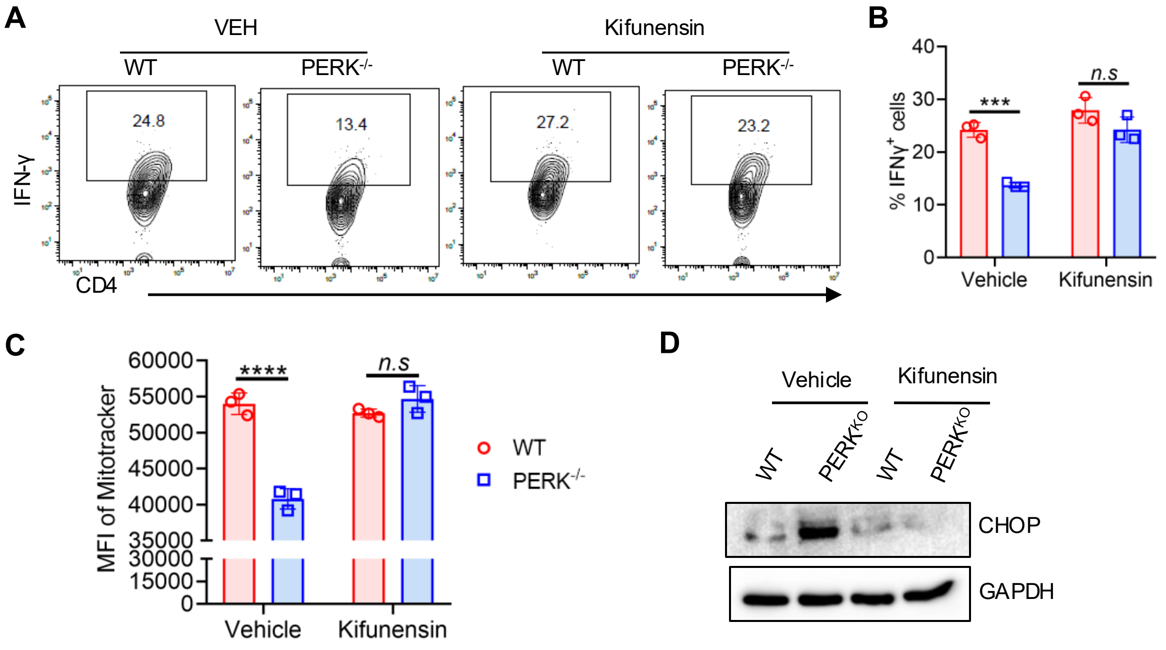

Supplemental Figure 14. Targeting ERAD inhibits T cell allogenic responses. (**A-D**) T cells isolated from B6 WT or PERK cKO mice were stimulated with allogeneic APCs from BALB/c mice for 4 days and treated with or without Kifunensin. (**A**) The levels of IFNγ in $CD4^+$ T cells were analyzed by flow cytometry. (**B**) Percentage of $IFN\gamma^+CD4^+$ T cells among gated $H2K^{b+}CD4^+$ T cells is shown. (**C**) MFI of Mito-tracker among gated $H2K^{b+}CD4^+$ T cells was evaluated by flow cytometry after incubating with Mito-tracker green. (**D**) Protein levels of CHOP and GAPDH in allogeneic T cells were evaluated by western blot. Data in panels **B**, **C** are represented as mean ± SD with biological replicates; significance was analyzed using a two-way ANOVA test. **P* < .05, ***P* < .01, ****P* < .001, *****P* < .0001.

Supplemental Figure 15

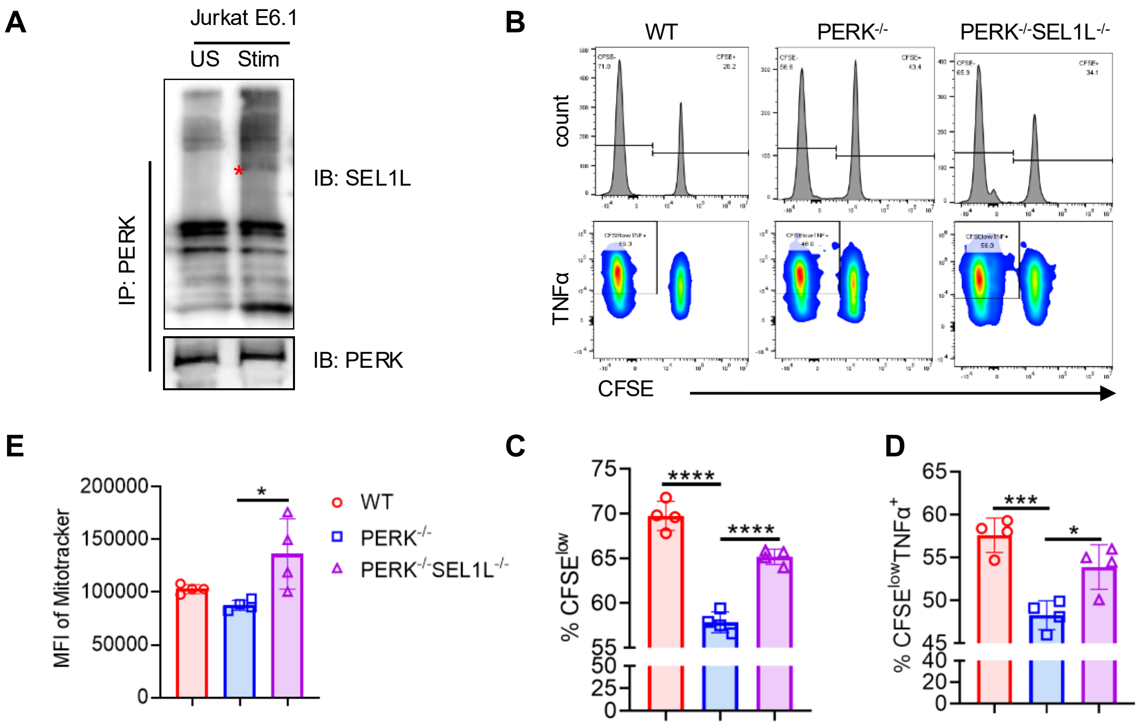

Supplemental Figure 15. PERK interacts with SEL1L, regulates T-cell allogeneic responses through ERAD. (**A**) Jurkat cells were stimulated with or without anti-CD3/TCR/CD2 complex (25 µl/ml) for 48 hours, then cells were lysed and immunoprecipitated with anti-PERK antibody and evaluated SEL1L by western blot. (**B-D**) T cells isolated from B6 WT or PERK cKO or PERK and SEL1L dKO mice were labeled with CFSE and stimulated with allogeneic APCs from BDF1 mice for 4 days. $CFSE^{low}$ and $CFSE^{low}TNF\alpha^{+}$ cells in $CD4^{+}$ T cells were analyzed by flow cytometry (**B**). Percentages of $CFSE^{low}$ (**C**) or $CFSE^{low}TNF\alpha^{+}$ (**D**) among gated $H2K^{d-}CD4^{+}$ T cells are displayed. (**E**) T cells isolated from WT B6 or PERK cKO or PERK and SEL1L dKO mice were stimulated with allogeneic APCs for 4 days. MFI of Mito-tracker among gated $H2K^{d-}CD4^{+}$ T cells was evaluated by flow cytometry after incubating with Mito-tracker green. Data in panels **C**, **D**, **E** are represented as mean ± SD with biological replicates; significance was analyzed using a one-way ANOVA test. **P* < .05, ***P* < .01, ****P* < .001, *****P* < .0001.

Supplemental Figure 16

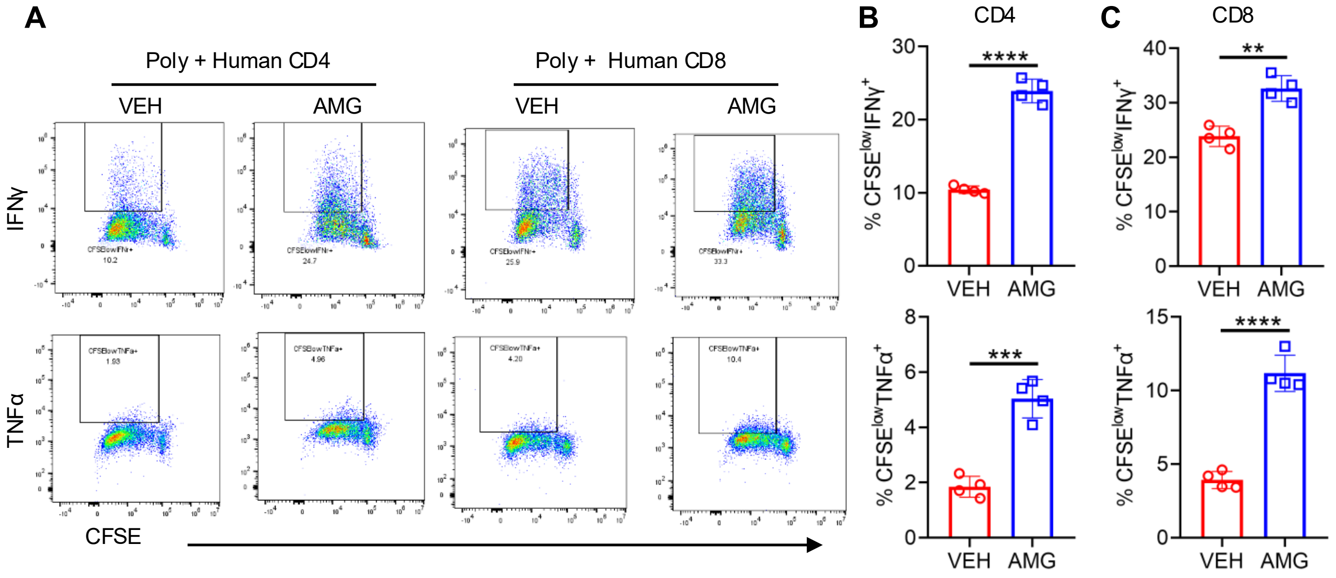

Supplemental Figure 16. Blockade of PERK increases human T-cell responses *in vitro.* (**A-C**) T cells isolated from human PBMCs were labeled with CFSE, stimulated with anti-CD3/TCR/CD2 complex (25 µl/ml) for 4 days and treated with or without AMG44. (**A**) CFSE^{low}IFNγ^{+}, CFSE^{low}TNFα^{+} cells in CD4^{+} or CD8^{+} T cells were analyzed by flow cytometry. (**B**) Percentages of CFSE^{low}IFNγ^{+}, CFSE^{low}TNFα^{+} cells among gated CD4^{+} T cells are displayed. (**C**) Percentages of CFSE^{low}IFNγ^{+}, CFSE^{low}TNFα^{+} cells among gated CD8^{+} T cells are displayed. Data in panels **B**, **C** are represented as mean ± SD with biological replicates, significance was determined using a two-tailed unpaired Student's *t* test. *$P < .05$, **$P < .01$, ***$P < .001$, ****$P < .0001$.
