# Supplementary material for: ER stress sensor PERK promotes T cell pathogenicity in GVHD by regulating ER-associated degradation
Source: J Clin Invest. 2025 Sep 30;135(23):e190958. doi: 10.1172/JCI190958 (PMC12646671; doi:10.1172/JCI190958)
Supplement: Unedited blot and gel images [file jci-135-190958-s247.pdf]

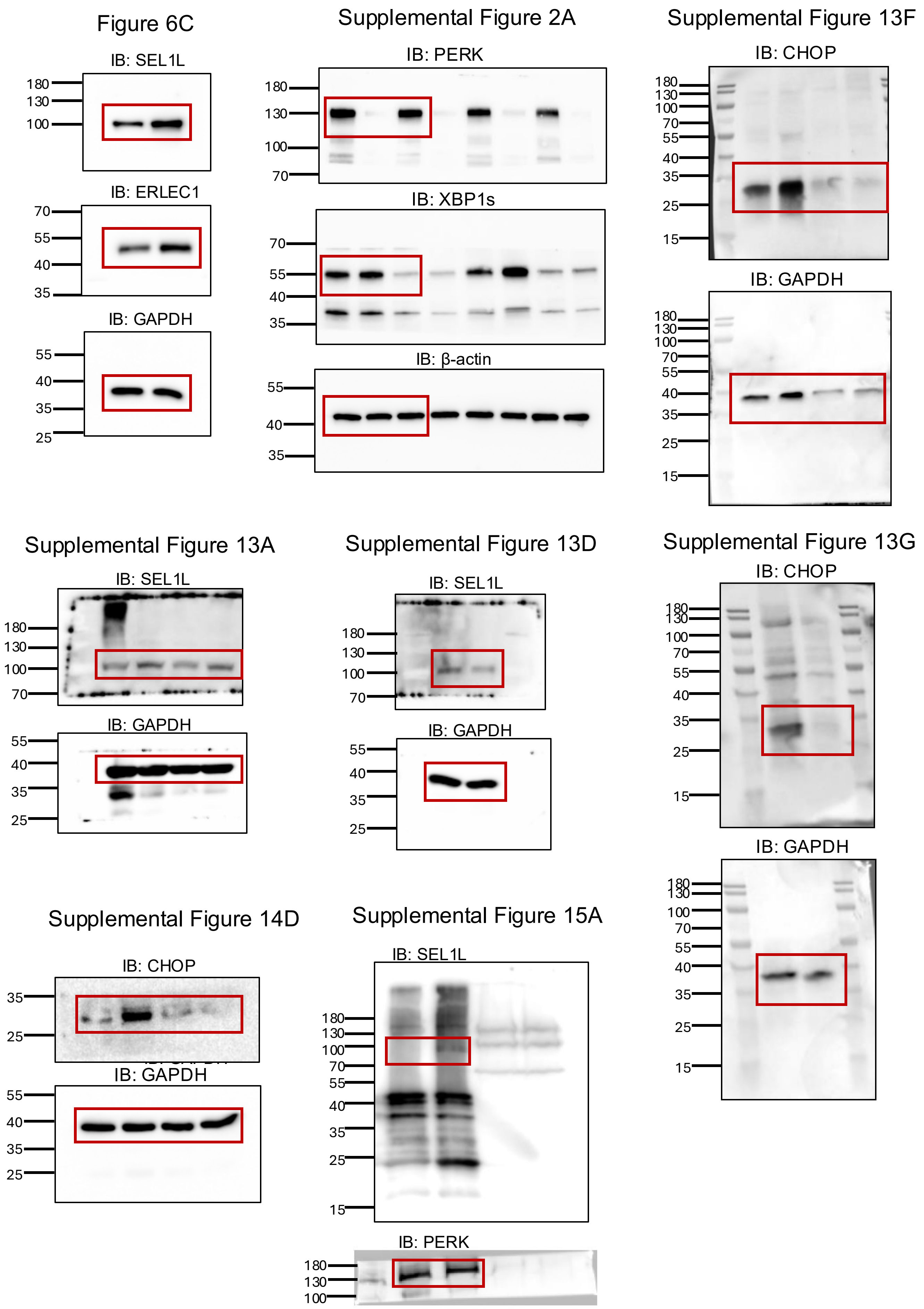
Figure 6C
IB: SEL1L
180
130
100
IB: ERLEC1
70
55
40
35
IB: GAPDH
55
40
35
25
Supplemental Figure 2A
IB: PERK
180
130
100
70
IB: XBP1s
70
55
40
35
IB: β-actin
55
40
35
Supplemental Figure 13F
IB: CHOP
180
130
100
70
55
40
35
25
15
IB: GAPDH
180
130
100
70
55
40
35
25
15
Supplemental Figure 13A
IB: SEL1L
180
130
100
70
IB: GAPDH
55
40
35
25
Supplemental Figure 13D
IB: SEL1L
180
130
100
70
IB: GAPDH
55
40
35
25
Supplemental Figure 13G
IB: CHOP
180
130
100
70
55
40
35
25
15
IB: GAPDH
180
130
100
70
55
40
35
25
15
Supplemental Figure 14D
IB: CHOP
35
25
IB: GAPDH
55
40
35
25
Supplemental Figure 15A
IB: SEL1L
180
130
100
70
55
40
35
25
15
IB: PERK
180
130
100
